# A willow sex chromosome reveals convergent evolution of complex palindromic repeats

**DOI:** 10.1186/s13059-020-1952-4

**Published:** 2020-02-14

**Authors:** Ran Zhou, David Macaya-Sanz, Craig H. Carlson, Jeremy Schmutz, Jerry W. Jenkins, David Kudrna, Aditi Sharma, Laura Sandor, Shengqiang Shu, Kerrie Barry, Gerald A. Tuskan, Tao Ma, Jianquan Liu, Matthew Olson, Lawrence B. Smart, Stephen P. DiFazio

**Affiliations:** 1 Department of Biology, West Virginia University, Morgantown, WV 26506-6057 USA; 2 Horticulture Section, School of Integrative Plant Science, Cornell University, New York State Agricultural Experiment Station, Geneva, NY 14456 USA; 3 HudsonAlpha Institute of Biotechnology, Huntsville, AL USA; 4 Department of Energy Joint Genome Institute, Walnut Creek, California USA; 5 Arizona Genomics Institute, School of Plant Sciences, University of Arizona, Tucson, AZ USA; 6 Biosciences Division, Oak Ridge National Laboratory, Oak Ridge, TN 37831 USA; 7 DOE-Center for Bioenergy Innovation (CBI), Oak Ridge National Laboratory, Oak Ridge, TN 37831 USA; 8 Key Laboratory of Bio-Resource and Eco-Environment of Ministry of Education, College of Life Sciences, Sichuan University, Chengdu, 610065 China; 9 State Key Laboratory of Grassland Agro-Ecosystem, Institute of Innovation Ecology & College of Life Sciences, Lanzhou University, Lanzhou, 730000 China; 10 Department of Biological Sciences, Texas Tech University, Box 43131, Lubbock, TX 79409-3131 USA

**Keywords:** Sex, Gene conversion, W chromosome, Palindrome, Genome, *Salix*

## Abstract

**Background:**

Sex chromosomes have arisen independently in a wide variety of species, yet they share common characteristics, including the presence of suppressed recombination surrounding sex determination loci. Mammalian sex chromosomes contain multiple palindromic repeats across the non-recombining region that show sequence conservation through gene conversion and contain genes that are crucial for sexual reproduction. In plants, it is not clear if palindromic repeats play a role in maintaining sequence conservation in the absence of homologous recombination.

**Results:**

Here we present the first evidence of large palindromic structures in a plant sex chromosome, based on a highly contiguous assembly of the W chromosome of the dioecious shrub *Salix purpurea*. The W chromosome has an expanded number of genes due to transpositions from autosomes. It also contains two consecutive palindromes that span a region of 200 kb, with conspicuous 20-kb stretches of highly conserved sequences among the four arms that show evidence of gene conversion. Four genes in the palindrome are homologous to genes in the sex determination regions of the closely related genus *Populus*, which is located on a different chromosome. These genes show distinct, floral-biased expression patterns compared to paralogous copies on autosomes.

**Conclusion:**

The presence of palindromes in sex chromosomes of mammals and plants highlights the intrinsic importance of these features in adaptive evolution in the absence of recombination. Convergent evolution is driving both the independent establishment of sex chromosomes as well as their fine-scale sequence structure.

## Introduction

Sex chromosomes carry genes that confer or control sex-specific traits [1]. In theory, the heterogametic (sex-specific) sex chromosome evolved from an autosome. There are two important features in sex determination regions (SDRs): suppressed recombination and the presence of sequences that only occur in one sex [1]. Furthermore, many sex chromosomes have lost most of their original genes over evolutionary time and accumulated repetitive sequences such as transposable elements and tandem gene duplications [2, 3]. Consequently, sex chromosomes can be difficult to sequence because they are often highly heterochromatic and have a large amount of repetitive and ampliconic DNA [1, 4].

A striking characteristic of mammalian sex chromosomes is the presence of large palindromes in ampliconic regions of the X and Y chromosomes that consist of large inverted repeats with highly identical sequences that are undergoing gene conversion [5, 6]. Ampliconic sequences on the human Y chromosome were acquired through transpositions from diverse sources, and then amplified [4]. These ampliconic sequences account for about 30% of the Y euchromatin [4]. The human Y chromosome palindromes contain eight gene families that are expressed predominantly in the testes and which are essential for spermatogenesis [6–8]. These genes undergo extensive gene conversion and have high sequence identity among the copies [6]. Other palindromes occur in the genome, but those on the sex chromosomes are by far the largest and have the highest rates of gene conversion [6, 9]. Palindromes have also been found on the W chromosomes of New World sparrows and blackbirds, suggesting that this may be a widespread feature of sex chromosomes [10]. However, such structures have not yet been described in plants.

Unlike in most animals, there is a lack of obvious sex chromosome heteromorphism in most dioecious plant species (i.e., differences are not readily discernable by cytology) [11, 12]. Sex determination systems are quite diverse in plants, and the mechanisms of sex determination have been identified for an increasing number of species in recent years [13]. For example, Y chromosomes have been intensively studied in papaya and persimmon. Both of these contain a female suppressor on the Y chromosome [13–15]. Recently, a female suppressing gene in asparagus has been identified on the Y chromosome using long-read sequencing technology with optical mapping [16]. Another study on octoploid strawberry found repeated transpositions of a female-specific gene cassette [17]. The genus *Silene* does have clearly heteromorphic sex chromosomes and has been a long-standing model for sex determination in XY plants. Female-suppressing and male-promoting factors were identified in *Silene* in the 1950s using genetic approaches [18]. More recently, it has been shown that some species of *Silene* have ZW sex determination systems, though it remains unclear if there are commonalities in the underlying mechanisms of sex determination in XY and ZW species [19].

Sex determination is similarly diverse within the Salicaceae family. SDRs have been consistently found on chromosome 15 with female heterogamety in multiple *Salix* species [20–22]. This is quite different from the closely related genus *Populus* where sex-determining regions consistently occur on chromosome 19, with most species showing male heterogamety [23, 24]. Previously, we reported that the SDR occupies a large portion of the W chromosome in *S. purpurea* with suppressed recombination extending over ~ 5 Mb [20, 25]. This is substantially larger than the SDR in *P. trichocarpa* and *P. balsamifera*, which appears to be approximately 100 kb in size [24, 26]. However, due to the structural complexity of the SDRs, none of these studies have thus far included an in-depth analysis of the sequence composition and structure of the SDRs, and it is unclear whether there is a common underlying mechanism of sex determination. Here we present a much more complete assembly of the *S. purpurea* W chromosome and report for the first time in plants a palindromic repeat structure that is similar to the one found on mammalian Y chromosomes. We also demonstrate that gene content is expanded on the W chromosome, and homologous genes occur in the *Salix* and *Populus* SDRs, suggesting that there may be some overlap in the underlying mechanisms of sex determination in this family.

## Results

### Genome assembly

We present here highly contiguous genome assemblies of a female and a male *S. purpurea*. The female assembly (94006 v4) consists of 452 contigs with an N50 of 5.1 Mb, covering a cumulative total of 317.1 Mb. Similarly, the male assembly (Fish Creek v3) has 351 contigs and an N50 of 5.6 Mb, covering 312.9 Mb (Additional file 1: Table S1). Both assemblies are partially phased in genomic regions where the two haplotypes are divergent. Alternative haplotypes are represented by 421 contigs totaling 72.4 Mb in the female assembly, and 497 contigs totaling 149 Mb for the male. Using a genetic map from a large intercross family derived from progeny of the sequenced male genotype, we created assemblies representing the 19 chromosomes, containing 108 contigs totaling 288.3 Mb for the female and 96 contigs totaling 288.5 Mb for the male. These represent over 90% of the assembled sequence in both cases, though 344 and 255 contigs remained unplaced by the genetic map for the female and male, respectively (Additional file 1:Table S2). The mapped and unplaced contigs are hereafter collectively referred to as the main genome, which excludes the alternative haplotypes.

Because we expected the W haplotype to be differentiated from the Z haplotype in the SDR, we anticipated that much of this region would be assembled as separate contigs. These can be readily differentiated by examining the relative depth of coverage when aligning male versus female short-read sequences against these references. After identifying the location of the SDR based on the presence of sex-linked markers [20], the initial Chromosome 15 assembly appeared to consist of a mix of Z and W scaffolds in a region we infer to be within the SDR (Additional file 2: Figure S1a). We therefore sought to create a new assembly with Z and W haplotypes assembled to separate chromosomes. To do this, we first identified the putative W contigs using sex association in a population of 60 unrelated individuals and differential depth of coverage in males and females from an F_2_ pedigree as criteria [20]. This resulted in identifying 23 contigs that were putatively comprised primarily of sequence derived from the W haplotype (Additional file 1: Table S3). One scaffold was excluded because it mostly consisted of an alternative haplotype of a longer contig of Chr15W.

Many of these contigs lacked markers from the intercross map that was used in the original genome assembly [20], particularly for those that came from portions of the W haplotype that were absent from the Z chromosome. We therefore created new genetic maps that had a mix of SNP and indel markers that would be more suited to capturing these hemizygous portions of the genome. The new genetic maps converged to 19 major linkage groups representing the 19 chromosomes. The male backcross map contained 8715 markers, while the female backcross map contained 8560 markers (Additional file 1: Table S4). We used these to assemble a Z and a W version of Chr15 (Additional file 1: Table S5). Thus, the current assembly (release ver5) contains 20 chromosomes, including Chr15Z and Chr15W. A total of 6.56 Mb (95.7%) of the W-specific contig sequence, contained in 17 contigs, was assembled to Chr15W using these maps. Four putative W scaffolds totaling 297 kb in length lacked mapped markers and could not be placed unambiguously.

### Location of the SDR

**Fig. 1:**
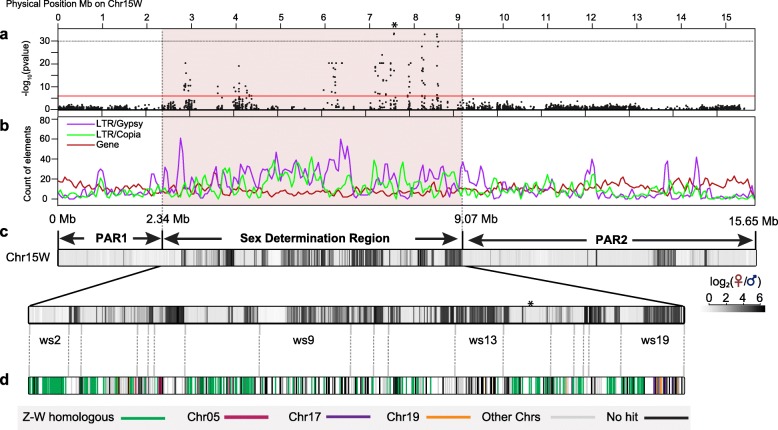
Genomic content of Chr15W and composition of the sex determination region (SDR). **a** A Manhattan plot of Chr15W, based on GWAS using SNPs derived from aligning to a reference genome lacking Chr15Z. The *Y* axis is the negative logarithm of *p* values, and the red line indicates the Bonferroni cut off. **b** Count of LTR elements including Gypsy and Copia, as well as genes in 100-kb windows with a 50-kb step size. **c** Distribution of female-biased sequence on Chr15W, along with a more detailed view of the SDR below. Each colored block shows the log_2_ of the ratio of female and male depth in 10-kb windows. Vertical gray lines below the figure show the boundaries of the contigs in the SDR. **d** Each tick represents a gene in the SDR. Colors indicate putative origins of the genes based on blastp versus the rest of the genome

We repeated sex association analysis for the 60 unrelated individuals using our new assembly with Chr15Z removed. Among 54,959 tested Genotyping by Sequencing (GBS) SNPs, all 105 significantly sex-linked SNPs were present only on Chr15W (Fig. 1a; Additional file 2: Figure S2a-c), and markers from PARs and other scaffolds in the main genome did not show any sex association (Additional file 2: Figure S2a). The eight top-ranking sex-associated markers were distributed from 7.66 to 8.66 Mb. Sex-associated markers were primarily heterozygous in females and homozygous in males, confirming our previously reported observation of ZW sex determination in *S. purpurea* [20].

### Composition of chromosomes 15 W and 15Z

Chr15W is 15.7 Mb in length, composed of 22 contigs placed with the new genetic map. For comparison, Chr15Z is only 13.3 Mb and is comprised of 16 contigs (Additional file 1: Table S5; Fig. 1). There are two pseudoautosomal regions (PARs), one at each end of Chr15W, that are indistinguishable from the corresponding regions on Chr15Z. PAR1 is 2.3 Mb long and is composed of one contig, and PAR2 is 6.5 Mb and is comprised of three contigs (Fig. 1). These regions are unphased and are therefore identical in the two assemblies.

The W-linked sex-determining region (SDR) is 6.8 Mb in length and occupies nearly 40% of the chromosome (hereafter referred to as the W-SDR). This region undergoes minimal recombination in the mapping population (Additional file 2: Figure S4). Reexamining male and female depth of coverage of the W-SDR, it is clear that this region of the genome is mostly phased to separate the male and female haplotypes (Additional file 2: Figure S1b). The region corresponding to the W-SDR on Chr15Z is only about 4 Mb in length, and only occupies 28.2% of the chromosome (hereafter referred to as the Z-SDR) (Additional file 2: Figure S3). Based on the ratio of male and female depth of coverage, the Z-W homologous regions that are present on both the Z and W chromosome are about 3.5 Mb and insertions that are unique to the W are about 3.1 Mb in the W-SDR (Fig. 1c).

The W-SDR has lower gene density and higher repeat density than other portions of the genome, suggesting that repetitive elements have accumulated in this region (Table 1). More specifically, both the W-SDR and the Z-SDR show lower gene density on average than the PARs or other autosomes. Similarly, both the W-SDR and Z-SDR show higher accumulation of Gypsy retrotransposons. Interestingly, Copia-LTRs occur at higher density in the W-SDR region compared to the Z-SDR (10.9% of W-SDR vs 5.9% of Z-SDR), (Kruskall-Wallis test, *P* < 2.2e−16) (Table 1), suggesting that these inserted following cessation of recombination between these haplotypes.

**Table 1:** Cumulative size in megabase of genes and LTR retrotransposons in different areas of the genome. Numbers in parentheses are percentages of the proportion of the specific type of regions

Category	W-SDR	Z-SDR	PAR	Autosomes*
Genes	1.56 (23.8)	1.14 (26.8)	3.72 (41.9)	104.31 (38.1)
Total repeats	3.16 (48.1)	1.81 (42.4)	2.58 (29.0)	89.17 (32.6)
Gypsy-LTR	0.86 (13.2)	0.55 (12.8)	0.38 (4.3)	15.45 (5.6)
Copia-LTR	0.72 (10.9)	0.25 (5.9)	0.37 (4.1)	13.87 (5.1)

*All 18 chromosomes are included

### Gene content of the W chromosome

**Fig. 2:**
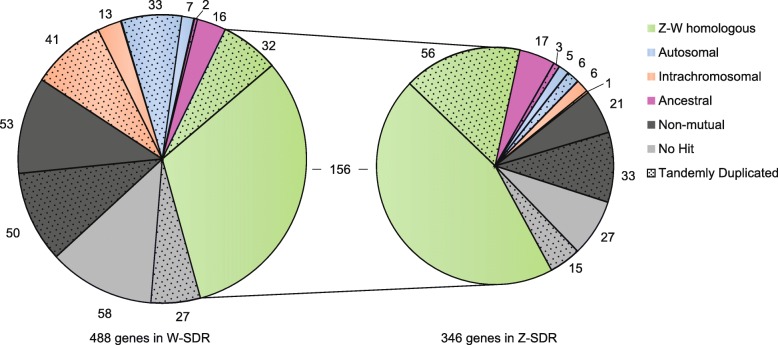
Annotated genes in Chr15W and Chr15Z. Genes are grouped according to the best non-self-hit in the annotated genome. Twenty-nine genes from an unmapped Z, scaffold_844 are also included. Stippled areas indicate genes of groups identified as tandem duplicates

There are 269 genes in PAR1, 778 genes in PAR2, and 488 genes in the W-SDR. In contrast, the Z-SDR only contains 317 genes (Fig. 2; Additional file 1: Table S6-S7). An additional 29 genes are present on scaffold_844, which is likely derived from the Z haplotype, but which lacked genetic markers to properly place it. To evaluate the completeness of the Z chromosome, we compared the gene content of this region to that from the Fish Creek male reference genome. The Z-SDR region was comprised of four contigs spanning from 2.86 to 7.10 Mb in Fish Creek, containing a total of 333 genes. Since the size and gene content were very similar between the Z chromosomes of the male and female references, we are restricting our analysis to the female to simplify the comparison.

There were 156 single-copy mutual best hits between the W-SDR and Z-SDR, referred to hereafter as Z-W homologs (analogous to X-degenerate genes on mammalian sex chromosomes) (Fig. 2). The W-SDR also contains 32 genes in tandem duplications, while the corresponding tandem repeats in the Z-SDR contain 56 genes. Additionally, the W-SDR contains 40 genes that have mutual best hits on other autosomes, and 33 of these are tandemly duplicated in the SDR. In contrast, the Z-SDR region contains only 11 such genes, only six of which are tandemly duplicated. These putatively transposed genes comprise 8% of the W-SDR and only 3% of the Z-SDR. Another 54 genes in the W-SDR resulted from intrachromosomal transpositions and subsequent tandem duplication, while only 7 genes in this category are found on the Z-SDR. In total, these transposed and ampliconic genes account for more than half of the discrepancy in gene content between the haplotypes. An additional 103 genes in the W-SDR had a top hit to other genes in the genome, but the best hit was not mutual, so these are lower confidence candidates for transpositions or Z-W homologs. The Z-SDR contained 54 such genes. The remaining genes had no significant hits to other genes in the genome, presumably due to loss by deletion, or gaps in the sequence or annotation (85 in the W-SDR and 42 in the Z-SDR).

### Z-W homologs and strata

**Fig. 3:**
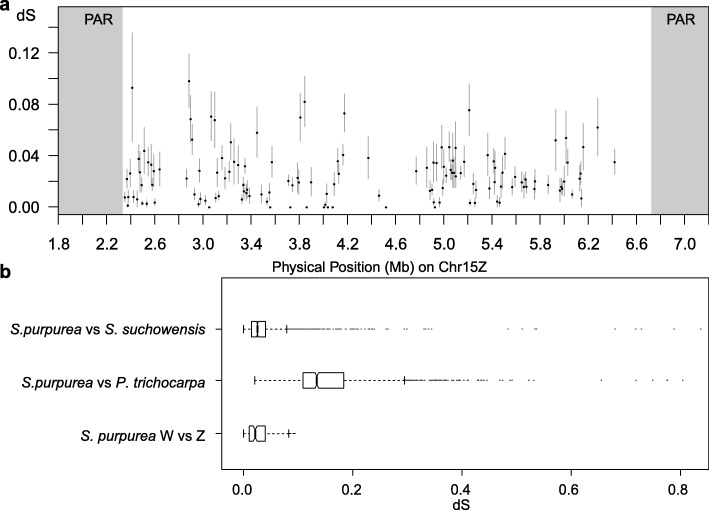
Synonymous substitution rates (*d*_S_) for genes in the SDR. **a** Comparison of syntenic genes in the W-SDR and Z-SDR. Bars represent standard errors. **b** Boxplot showing distributions of interspecific synonymous substitutions for 1365 syntenic genes on Chr01 for the closely related species *S. purpurea* and *S. suchowensis* and for 1363 genes on Chr01 in *S. purpurea* and *Populus trichocarpa*, compared to the distribution of substitutions between syntenic genes in the *S. purpurea* SDR

We used syntenic gene pairs identified through MCScanX between the W-SDR and Z-SDR to test if there are strata with different degrees of divergence based on synonymous substitutions (*d*_S_), which would indicate different phases of cessation of recombination [27]. There was little evidence to support the presence of strata based on 156 pairs of Z-W homologs (Fig. 3 and Additional file 1: Table S8). The average *d*_S_ was 0.027 ± 0.020 SE. For comparison, the *d*_S_ between syntenic genes on Chr01 for *S. purpurea* and *S. suchowensis* was 0.045 ± 0.0022 SE, and the *d*_S_ between *S. purpurea* and *P. trichocarpa* was 0.146 ± 0.0022 SE for syntenic genes on Chr01 (Fig. 3).

### Transpositions to the W-SDR and palindromic repeats

**Fig. 4:**
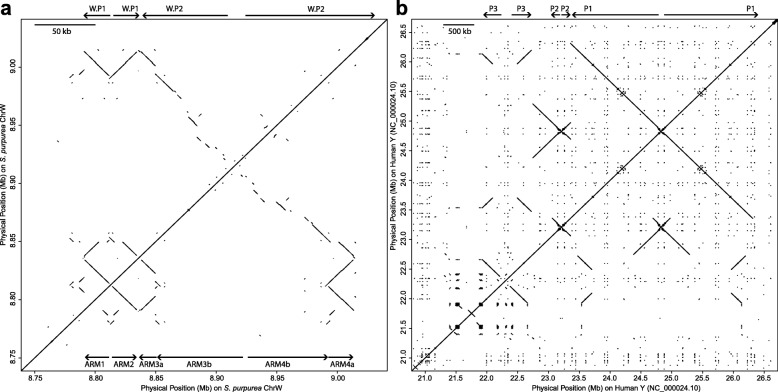
Palindromic repeats in the *S. purpurea* W chromosome (**a**) and the *H. sapiens* Y chromosome (**b**). The dot plots were produced using LASTZ with identical settings. Note the different scales, indicated by the bar at the top right of each figure. *H. sapiens* palindromes are labeled following Skaletsky et al. [4]

The recently transposed genes are of particular interest because they could provide a potential mechanism for establishment of the SDR and could highlight genes that are potential candidates for sex determination and/or sex antagonism [28]. Among 40 genes putatively transposed from autosomes to the W-SDR, 7 have best hits on Chr19 (manually annotated genes excluded) (Additional file 1: Table S9). Contig ws19 is particularly enriched for transposed genes and merits a closer examination (Fig. 1). Contig ws19 contains 11 transposed genes, including four genes from Chr19 and four genes from Chr17 (Fig. 1). Many of these transposed genes occur in two to four copies on ws19 in striking inverted repeat configurations that are similar to the palindromic repeats that occur on mammalian Y chromosomes (Fig. 4).

**Fig. 5:**
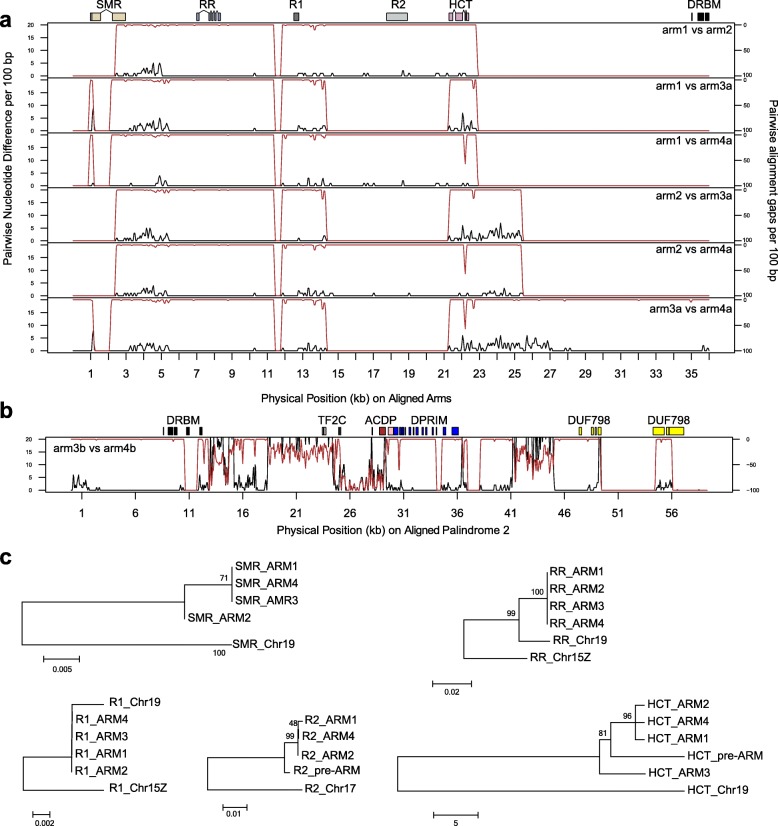
Sequence comparisons for the two palindromes. **a** Comparison of the four arms that are shared among the two palindromes. The black line represents the number of nucleotide differences in 100-bp windows, while the red line indicates gaps in the alignment on an inverted scale. **b** Comparison of the portions of palindrome 2 that are not shared with palindrome 1. **c** Phylogenetic trees of five multi-copy genes in the palindromic region

In *S. purpurea*, this region is female-specific (i.e., it occurs in all females but in no males) and is composed of two palindromes. Palindrome W.P1 spans about 42.7 kb with a 2.6-kb spacer in the center, and Palindrome W.P.2 is immediately adjacent and spans over 165 kb (Table 2; Fig. 4a). A 20-kb sequence occurs in inverted orientation and shows high sequence identity across the four arms of both palindromes (Table 2; Fig. 5a). In palindrome W.P1, these are referred to as arm1 and arm2, and in Palindrome W.P2, these are referred to as arm3a and arm4a (Table 2; Fig. 4a). Sequence identity among these four arms is greater than 99% on average. The regions of high sequence identity are disrupted by a ~ 500 bp insertion in the center of arm4. Furthermore, arm3 has a 6.9 kb deletion at 11.7 kb, followed by a stretch of 1.6 kb that can be aligned to the other arms in the same orientation (Fig. 5a). Additionally, there is a 12-kb stretch upstream of arm1 that shows high identity to portions of arms 1 and 2. We call this the pre-arm for convenience (Table 2).

**Table 2:** Coordinates of palindromes in the female SDR

	Name	Start (bp)	End (bp)	Size (bp)	Gene families
	Pre-arm	8,778,973	8,791,042	12,070	*R2,HCT*
Palindrome W.P1	arm1	8,790,932	8,811,002	20,071	*SMR,RR,R1,R2,HCT*
	Spacer1	8,811,003	8,814,588	3586	
	arm2	8,814,589	8,834,138	19,550	*SMR,RR,R1,R2,HCT*
Palindrome W.P2	arm3a	8,836,813	8,850,772	13,960	*SMR,RR,R1,HCT*
	arm3b	8,850,773	8,920,527	69,755	*DRBM,TF2C,DPRIM,DUF789*
	Spacer2	Unidentified			
	arm4b	8,920,528	8,993,098	72,571	*DRBM,ACDP,DPRIM,DUF789*
	arm4a	8,993,099	9,013,390	20,292	*SMR,RR,R1,R2,HCT*

Palindrome W.P2 contains an additional inverted repeat that is missing from W.P1. We refer to this as arm3b and arm4b (Table 2; Fig. 4a). Sequence identity is somewhat lower between these two arms compared to the other four, ranging from 96 to 99% over most of their length. Furthermore, the regions of high identity are disrupted by numerous insertions and deletions (Fig. 5b).

### Gene content of the palindromes

There are five genes duplicated across arms 1, 2, 3a, and 4a of both palindromes. These are the Small Muts-Related protein (SMR), a Type-A cytokinin response regulator (RR), two genes that contain an NB-ARC domain (R1 and R2), and a hydroxycinnamoyl-CoA shikimate/hydroxycinnamoyl transferase (HCT) (Table 3). All of these genes except R2 have clear paralogous copies on Chr19. There is very little sequence divergence among most of these paralogs in the palindromes (Fig. 5).

**Table 3:** Genes present in palindromes 1 and 2

	Gene symbol	Number of copies	GeneID	Chromosome of the non-W best hit	Best hit in *A. thaliana*	Arabidopsis name or description (function)	Best hit in *P. trichocarpa* v3	Identity *of P. trichocarpa* best hit
Palindromes W.P1 and W.P2	*SMR*	4^[a]^	Manually annotated	Chr19	AT5G23520	*SMR* (Small MutS Related) domain-containing protein)	Potri.T013000	90.70
	*RR*	4	Sapur.15WG073500	Chr19	AT3G56380	*ARR17* (type A cytokinin response regulator)	Potri.019G133600	92.81
			Sapur.15WG073900					
			Sapur.15WG074000					
			Sapur.15WG075200					
	*R1*	4^[a]^	Sapur.15WG073800	Chr15Z	AT4G27220	NB-ARC domain-containing disease resistance protein	Potri.T012900	81.00
			Sapur.15WG074100					
	*R2*	3(1)^[a]^	Manually annotated	Chr17	AT4G27220	NB-ARC domain-containing disease resistance protein	Potri.T013300	61.23
	*HCT*	4(1)	Sapur.15WG073400	Chr19^[b]^	AT5G48930	*HCT* (hydroxycinnamoyl-coa shikimate/quinate hydroxycinnamoyl transferase)	Potri.018G104700	58.02
			Sapur.15WG073600					
			Sapur.15WG073700					
			Sapur.15WG074200					
			Sapur.15WG075100					
Palindrome W.P2 only	*DRBM*	2	Sapur.15WG074300	Chr17	AT1G09700	*ATDRB1* (dsRNA binding protein)	Potri.017G126700	61.95
			Sapur.15WG075000					
	*TF2C*	1	Sapur.15WG074400	Chr08^[c]^	AT2G27040	*AGO4* (*ARGONAUTE 4*, siRNA mediated gene silencing)	NA	NA
	*ACDP*	1	Sapur.15WG074900	Chr17	AT5G52790^[d]^	CBS domain protein with DUF21 (transmembrane transporter)	Potri.017G147900	83.33
	*DPRIM*	2	Sapur.15WG074500	Chr17	AT5G52800	DNA primase	Potri.017G148000	92.52
			Sapur.15WG074800					
	*DUF789*	2	Sapur.15WG074600	Chr17	AT1G03610	*DUF789* (protein of unknown function)	Potri.017G152600	86.03
			Sapur.15WG074700					

^[a]^Manually annotated transcripts were included in the count. Numbers in the parentheses are from a fragment in the upstream portion of W.P1 that is homologous to part of W.P1. ^[b]^This cluster of tandem duplications on Chr19 in *S. purpurea* is not present on Chr19 in *P. trichocarpa*. ^[c]^The palindrome gene contains only a truncated blast hit to Sapur.008G005800 on Chr08. ^[d]^This best hit with an expected value of 8 × 10^−3^ due to a sequence length of 84 aa. Expected values of the remaining *A. thaliana* were less than 1 × 10^− 10^

The cytokinin response regulator is of particular interest because an ortholog of this gene has also been found to be associated with sex in *Populus* [24] and is therefore an excellent candidate as a sex determination gene in the Salicaceae. The RR gene is highly conserved across all four palindrome arms on the W-SDR (Fig. 5a, c). Interestingly, we also found a pseudogene copy of the RR gene on the Z-SDR. This is the only one of the five genes that is present in some form on the W-SDR, the Z-SDR, Chr19, and also in the SDR of *Populus*. There is a 2.6-kb sequence inserted upstream of all RR copies in the palindrome, and not in the Z-SDR pseudogene or on Chr19 (Additional file 2: Figure S5). This suggests that the W-SDR palindrome formed after transposition from Chr19. Interestingly, the RR gene also occurs as inverted repeats in all three locations in the genome (W-SDR, Z-SDR, and Chr19). However, alignment of the W-SDR, Z-SDR, and Chr19 versions demonstrates that the palindromes likely formed independently, because the palindromic regions are different (Additional file 2: Figure S5).

There are an additional five genes in the W.P2 palindrome. Three of these genes occur as inverted repeats: a DNA-directed primase/polymerase protein (*DRBM*), a DNA primase (*DPRIM*), and a protein containing Domain of Unknown Function 789 (*DUF789*). In addition, there is a homolog of ARGONAUTE 4 (*TF2C*) and a CBS domain protein (*ACDP*) in single copy. Four of these genes were apparently transposed from Chr17 (Table 3). This leads us to the hypothesis that after these genes were transposed to the W-SDR they underwent several rounds of structural rearrangements, including duplications, inversions, and deletions.

### Multiple LTR retrotransposons in the palindrome

**Fig. 6:**
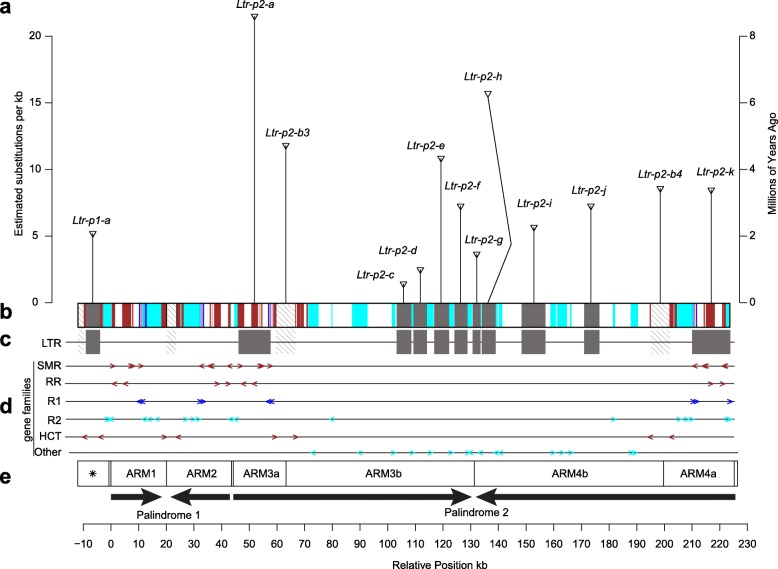
LTR retrotransposons, female-specific genes, and palindromes. **a** Each vertical line with a wedge on top represents each of the 13 TEs identified in the palindromic region by LTRharvest. The height of each line indicates the number of estimated nucleotide substitutions in the two LTRs (transposons a-h), and an approximation of the insertion time based on the mutation rate in *P. tremula* [29]. **b** Colored boxes represent putative chromosomal origins of genes in the palindrome. Dark red, Chr19, cyan, Chr17. Blue boxes represent genes with paralogs on the Z chromosome. **c** The positions of 13 LTRs (shaded boxes). Hatched boxes represent incomplete duplications derived from *Ltr-p2-b3/b4*. **d** Exon positions and orientations, represented by colored arrows. **e** Schematic representation of female-specific palindromes. The box with a star represents a homologous region derived from part of one of the arms (preARM). Directions of arrows indicate the relative orientations of the four arms

To gain further insight into the composition and history of the W-SDR, we used LTRharvest and LTRdigest to annotate LTR retrotransposons in the palindromic region. We identified one LTR retrotransposon in the pre-Arm region and 12 LTR retrotransposons in palindrome W.P2 that have terminal repeats identified with coding regions (Fig. 6a). These 13 retrotransposons are likely to be independent insertion events given that they have different long terminal repeats as well as different target site duplications and do not occur in the same position in the opposite arm of the palindrome (Additional file 1: Table S10). Given that there are varying numbers of substitutions within the LTRs of the same retrotransposon, it appears that these insertions have occurred repeatedly after establishment of the palindromes. Using a previous estimation of the mutation rate in *P. tremula* (2.5 × 10^− 9^ per year) [29], we estimate that the oldest insertion occurred at least 8.6 ± 2.9 s.d. MYA from a nonautonomous LTR retrotransposon, *Ltr-p2-a* (Fig. 6a and Additional file 1: Table S10). This is likely an underestimate, since the *Salix* substitution rate is substantially higher than that of *Populus* [30]. Since the oldest substitutions occurred in Palindrome W.P2, we infer that this element became established first (Fig. 6a). The LTRs of the nonautonomous elements *Ltr-p2-a* and *Ltr-p2-k* flank the *SMR* and *RR* genes (Fig. 6c, d; Additional file 2: Figure S6), which raises the intriguing possibility that these LTRs were involved in the transposition of these genes to this region. However, the target site duplications for these copies are identical across the palindrome arms, suggesting that the duplications and rearrangements of these genes in the W-SDR did not involve these elements (Additional file 2: Figure S6). We also found two highly similar LTRs from the same family in W.P1 (*Ltr-p2-b3* on arm3 and the *Ltr-p2-b4* on arm4; Fig. 6a–c; Additional file 1: Table S10). There are truncated parts of this LTR in the pre-arm and the spacer between arm1 and arm2 as well (Fig. 6b, c). These copies might be a direct consequence of duplications and inversions that occurred during the formation of the palindrome instead of independent insertions.

### Evidence for gene conversion in the palindromes

**Fig. 7:**
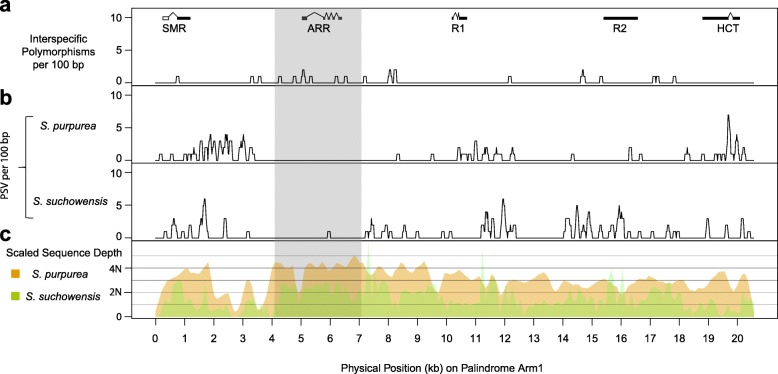
Sequence variation in the palindrome arms. **a** Density of fixed differences between *S. purpurea* and *S. suchowensis* per 100 bp. **b** Density of paralogous sequence variants (PSVs, differences among the four palindrome arms) in *S. purpurea* and *S. suchowensis*. **c** Relative depth of Illumina sequence reads aligned to a reference sequence of one arm of the *S. purpurea* palindrome, where 2N represents the expected depth of read alignment across the whole genome. The gray shaded area represents a segment of the palindrome that is enriched for interspecific fixed variants, but depleted in PSVs, providing strong evidence for differential gene conversion in the two lineages

We have shown that the palindromes are likely to be millions of years old based on the retrotransposon analysis, yet sequence identity of portions of the palindrome arms remains high (Fig. 5a). The most parsimonious explanation for this is gene conversion among the palindrome arms, as has been observed in the mammalian Y chromosome palindromes [6, 31]. To test for this, we searched for regions that had interspecific base substitutions relative to *Salix suchowensis*, a closely related species with ZW sex determination [22]. If regions with interspecific substitutions lack paralogous sequence variation (PSV) across the palindrome arms, then this would be excellent evidence of gene conversion [31]. We detected a 3-kb region within the palindromes where there are no PSVs in *S. purpurea* and only one PSV in *S. suchowensis*, but substantial interspecific polymorphisms (Fig. 7). The depth of this region is 4N as expected for the four copies of the palindrome arms in *S. purpurea*. In *S. suchowensis*, the depth is between 2N and 3N, which indicates that there might be a palindrome structure as well, though it might be incomplete. We also applied the same methods with resequencing reads of two female and two male *S. viminalis* individuals (another *Salix* with ZW sex determination) [21], but the palindromic region was not well covered by reads of either sex. This may indicate that *S. viminalis* lacks the palindrome, though it is more distantly related to *S. purpurea* than is *S. suchowensis*, so this may simply be due to excessive sequence divergence in this region.

### Expression patterns of genes in the palindromes

**Fig. 8:**
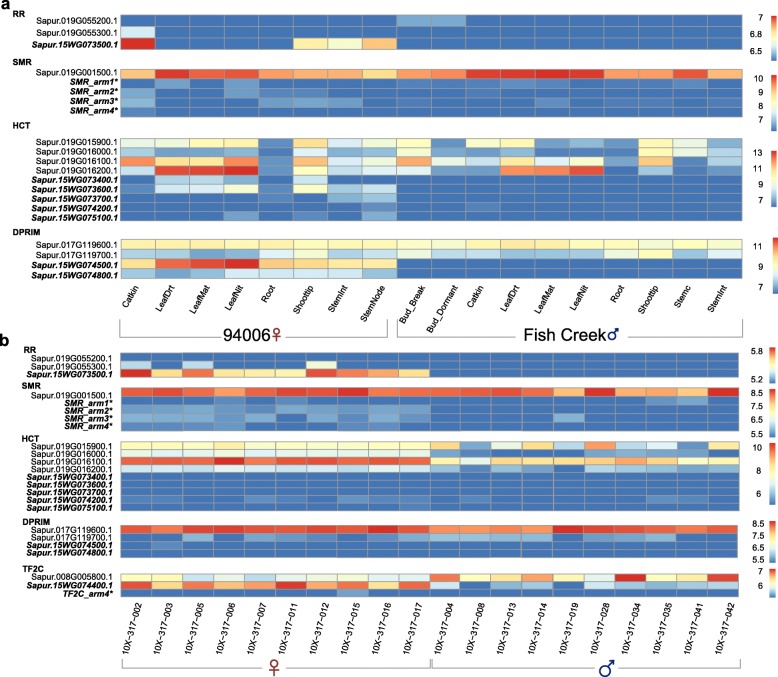
Expression profile of genes from the W palindromes and autosomal paralogs. **a** Normalized read counts of genes in different tissues from clone 94006 (female) and Fish Creek (male). **b** Normalized read counts of selected genes in catkins from 10 females and 10 males from an F_2_ family. Gene labels in bold font are from the palindromes. Asterisks indicate manually annotated genes

We examined expression profiles in multiple tissues of the two reference genomes to validate the predicted transcripts and to determine how the expression patterns of genes in the palindromes differ from their autosomal counterparts. Most genes in the palindromes show female-limited expression while the autosomal copies are generally not sex-biased (Fig. 8a). The cytokinin response regulator (*RR*) (Sapur.15 W073500) shows the highest expression in catkin tissue, followed by expression in shoot tips and stems. On the contrary, two autosomal copies on Chr19 show lower expression, limited to female catkins and male buds. The four copies of the *SMR* gene show low expression in female catkins and other tissues, but the autosomal copy on Chr19 (Sapur.019G001500) is expressed in all tissues (Fig. 8a). All five copies of the *HCT* gene from the palindromes showed low expression in female catkins and roots and higher expression in leaf tissues, shoot tips, and stems, all of which were female-biased. Two copies of the DNA Primase gene from palindrome W.P2 also show high expression in leaf tissues while the original copy on the autosome (Sapur.017G119600) was expressed across all sampled tissues. Similarly, analysis of transcriptomic data of catkins from 10 females and 10 males in the F_2_ family confirms that the genes in the palindromes are primarily expressed in female tissue, in contrast to their autosomal paralogs (Fig. 8b).

## Discussion

### The W chromosome in *S. purpurea*

Using depth of coverage for males and females from a controlled cross pedigree, we have been able to identify Z and W haplotypes from the SDR of a highly heterozygous species from a standard PacBio assembly. We also show how presence-absence markers generated from sequence depth in controlled cross progeny can be used to genetically map hemizygous portions of the SDR. In a similar study of a young Y chromosome in asparagus, BioNano optical maps for a YY individual were generated to improve genome contiguity, and sequence depth of coverage was also treated as a QTL to aid the assembly because of the presence of large indels in the sex chromosome [16]. Here, we showed that by combining long-read sequencing with GBS marker data from a large F_2_ family, we could efficiently identify the male and female haplotypes in the SDR. However, unlike strategies like single-haplotype iterative mapping and sequencing (SHIMS) that have been used in assemblies of mammalian Y chromosomes [4, 32–34], our map-based strategy could not provide a definitive order for the W contigs due to lack of recombination in the SDR.

The W-SDR is approximately 2.5 Mb larger than the Z-SDR. This is due in part to a greater accumulation of transposable elements, which account for approximately 1.35 Mb of this difference. This is consistent with expectations for sex chromosome evolution where transposable elements are expected to accumulate in regions with suppressed recombination [1, 35, 36]. However, gene content of the sex chromosome is expected to decrease due to the absence of recombination and reduced efficiency of purifying selection [1, 27]. Instead, we observed that gene content is expanded in the W-SDR, driven in part by numerous transpositions and subsequent expansion of autosomal genes. Autosomal transpositions have also been demonstrated in other sex chromosomes, including mammalian Y chromosomes [6]. The recently formed neo-Y chromosome of *Drosophila miranda* also shows massive expansion of genes that have been translocated from autosomes, and these are enriched for genes contributing to sex-specific functions [37].

Sex chromosomes commonly show evidence of “evolutionary strata” with markedly different levels of sequence divergence that represent different epochs of expansion of the SDR [35]. Under one common model of sex chromosome evolution, these strata are the result of multiple periods of SDR expansion as sexually antagonistic polymorphisms become incorporated into the SDR [27, 38]. Although the identified SDR in *S. purpurea* is about 6–7 Mb, occupying more than one third of the W chromosome assembly, we detected little evidence for the existence of such strata. This corroborates a previous analysis that failed to detect strata in *S. suchowensis* using an integrated segmentation and clustering method [39]. It appears that cessation of recombination has not been a gradual long-term process in the *S. purpurea* SDR, although it is certainly possible that the oldest strata have decayed to the point where they cannot be meaningfully aligned. An explanation for the large size of this region is that it partially overlaps with the centromere of Chr15, as we previously reported [20]. It is possible that the repressed recombination in this region pre-dated the transposition of a relatively small SDR cassette, as has been observed in octoploid *Fragaria* [17]. This is consistent with the apparently small size of the region in *Populus* (~ 100 kb), which is located on a different chromosome [24]. This is also consistent with the structure and composition of the palindromic repeats that we discovered in *S. purpurea*, which are excellent candidates as sex determination loci, as detailed below.

### Sex chromosome palindrome repeats

We have reported here the first observation of a large inverted repeat in a plant sex chromosome, similar to the palindromic structures observed in mammalian sex chromosomes. We have further demonstrated that these palindromes are undergoing gene conversion, suggesting functional similarities to mammalian sex chromosome palindromes. W.P1 and W.P2 of *S. purpurea* have a similar arrangement of arms as P1 and P3 in humans due to the presence of highly homologous regions between the two palindromes. Similar palindromes have been also been discovered on Y chromosomes of other mammals, as well as avian W chromosomes (reviewed by [5, 6]). Large mammalian palindromes developed as a series of accumulations of insertions from autosomes and maintained through arm-to-arm gene conversion. This intrachromosomal gene conversion can maintain coding sequence integrity which otherwise would be compromised by the continuous accumulation of deleterious mutations in the absence of homologous recombination (i.e., Muller’s ratchet) [5, 6, 31, 40]. The fact that these structures have independently evolved in non-recombining regions of sex chromosomes is an intriguing case of convergent evolution of chromosome structure. Interestingly, the chloroplast genome, another non-recombining chromosome in plants, also contains a different large inverted repeat that undergoes gene conversion [41] and helps maintain structural integrity of the genome, suggesting that this phenomenon may be common in regions of the genome that lack recombination [42]. However, it is also important to note that not all palindromic repeats occur in regions of the genome with suppressed recombination, most notably the large palindromes on the mammalian X chromosome. Palindromes may therefore play another role beyond maintenance of sequence integrity, such as mitigating expression of sexually antagonistic genes [9] or in gene dosage compensation in the heterogametic sex [43, 44].

The *S. purpurea* palindromes are considerably smaller than mammalian palindromes and have only accumulated two major autosomal transpositions (from Chr17 and Chr19), possibly reflecting their young age. Another difference between the human palindrome and the one in *S. purpurea* is that the gene conversion seems to be quite efficient across all the eight palindromes in humans, but the observed regions under gene conversion in *S. purpurea* are much more limited. This is particularly obvious in W.P2, compared to human P1, which has high sequence identity over several megabases (Fig. 4). Nevertheless, we found strong evidence for gene conversion in the cytokinin response regulator gene, based on an absence of PSVs. The ortholog of this gene in *S. suchowensis* has accumulated divergent nucleotide substitutions, which also seem to be homogenized among copies. This is a clear signature of gene conversion and is unlikely to result from purifying selection or very recent independent duplication events [31].

### Evidence for a possible shared evolutionary history for the *Populus* and *Salix* SDRs

Initial analyses in *P. trichocarpa* suggested that the SDR is much younger than the whole genome duplication event that is shared by *Populus* and *Salix*, suggesting that the SDR became established well after these genera diverged [24]. The low divergence between homologs in the fully sex-linked region (i.e., between Chr15W and Chr15Z homologs) shows that the SDR *of S. purpurea* evolved recently. Furthermore, given that the SDR is located in approximately the same portion of Chr15 in both *S. purpurea* and *S. suchowensis*, and both have ZW systems [20, 22], it is reasonable to assume that the SDR became established in this lineage prior to divergence of these two species, but well after divergence from *Populus*, which has an XY SDR on Chr19. On this basis, it has been hypothesized that these SDRs have independent evolutionary origins [22]. We believe that our results point toward a single origin of dioecy in these genera, as well as shared components of an underlying sex determination system focused on cytokinin-mediated regulation.

Support for this hypothesis is provided by the type A cytokinin response regulator homologs that occur in palindrome arms 1,2,3a, and 4a (Table 3), which show strong evidence of ongoing gene conversion and female-specific expression in *S. purpurea*. The best ortholog of these genes in *P. trichocarpa* is Potri.019G133600 (this gene was originally designated *PtRR11*, but it is referred to as *RR9* in subsequent publications [45–47], so we will adopt that nomenclature here to avoid confusion). *PtRR9* grouped with the *Arabidopsis thaliana* type A response regulators *ARR16* and *ARR17* in the original phylogenetic analysis of this family in *Populus* [48]. The *ARR16* gene has been implicated in gynoecial development in Arabidopsis [49]. *PtRR9* is expressed primarily in reproductive tissues in *Populus* [47, 48] and is also associated with sex in several *Populus* species [24, 45, 46]. Further supporting its possible role in sex determination, it was the only gene in the *P. balsamifera* genome that showed clear sex-specific differences in promoter and gene body methylation [45]. This raises the intriguing possibility the mechanisms of sex determination in ZW *Salix* and XY *Populus* share common regulatory elements and a shared evolutionary origin.

The cytokinin signaling pathway has emerged in recent years as a prominent candidate for regulating floral development and sex expression in plants [50, 51]. The potential role of cytokinin signaling in dioecy has recently been highlighted by the groundbreaking study by Akagi et al. in kiwifruit (*Actinidia* spp.) [51]. The authors identified a Type C response regulator (*Shy Girl, SyGI*) on the Y chromosome that was associated with maleness. Overexpression of this gene in Arabidopsis and *Nicotiana tabacum* caused suppression of carpel development, supporting its potential role as a suppressor of female function [13]. This work has some interesting parallels with the results reported here for *Salix* and *Populus*. First, type C response regulators are essentially similar in structure to Type A response regulators, with the main difference being that Type C is not induced by cytokinin. Interestingly, *PtRR9* also was not induced by exogenous cytokinin application [48], though this has not yet been tested with floral tissue. Second, *SyGI* was duplicated from an autosomal gene and subsequently gained a new function on the Y chromosome, much like *SpRR9* has been duplicated from Chr19 in *S. purpurea* and established a distinct pattern of expression, and presumably new functions. However, *RR9* and *SyGI* are clearly not orthologous and likely perform different roles in cytokinin signal transduction. This supports the view that there are numerous ways to achieve separate sexes in plants, and it is likely that a myriad of mechanisms underlie the hundreds of independent occurrences of dioecy in the angiosperms [52], even if a relatively small number of pathways are involved [13, 53].

## Conclusion

We have shown that the SDR on the W chromosome of *S. purpurea* has expanded gene content compared to the corresponding region on the Z chromosome, due in part to autosomal genes that have been transposed and expanded in the region of suppressed recombination. We further demonstrated that some of these transposed genes are arranged as palindromic repeats that are undergoing gene conversion, suggesting some functional similarities to the mammalian sex chromosomes. This is a striking example of convergent evolution in chromosome structure. We have also demonstrated that the coding sequence undergoing gene conversion in the palindrome, *SpRR9*, is orthologous to a gene that is also associated with sex in *Populus*. This gene is an excellent candidate for controlling sex determination through modulation of the cytokinin signaling pathway. However, much remains to be determined about the underlying mechanism of sex determination. Most importantly, it is currently unclear how the same gene is functioning in an XY system in *Populus* and a ZW system in *Salix*. It is possible that the W chromosome version acts as a dominant promoter of female function, while the Y version is a dominant suppressor of female function, based on the putative roles of cytokinin and the type A response regulators in female development in Arabidopsis. A detailed model should emerge through comparative analysis of the W and Y chromosomes of multiple species in the Salicaceae, which is currently underway. If the underlying mechanism shares common regulatory elements, this will be the first case demonstrating XY and ZW systems that are controlled by the same pathway in plants.

## Methods

### Initial assembly of the genome

Whole genome assemblies were produced for two *S. purpurea* clones: female clone 94006, and a male offspring of this clone, “Fish Creek” (clone 9882-34), which was derived from a controlled cross between clone 94006 and male *S. purpurea* clone 94001. Clones 94001 and 94006 were collected from naturalized populations in upstate New York, USA. Sequencing reads were collected using the Illumina and PACBIO platforms at the Department of Energy (DOE) Joint Genome Institute (JGI) in Walnut Creek, California, and the HudsonAlpha Institute in Huntsville, Alabama. Illumina reads were sequenced using the Illumina HISeq platform, and the PACBIO reads were sequenced using the RS platform. One 400 bp insert 2 × 250 Illumina fragment library was sequenced for total coverage of 183× in clone 94006 and 153× in Fish Creek. Prior to use, Illumina reads were screened for mitochondria, chloroplast, and ΦX174 contamination. Reads composed of > 95% simple sequence were removed. Illumina reads < 50 bp after trimming for adapter and base quality (*q* < 20) were removed. For the PACBIO sequencing, a total of 47 P6C4 chips (10 h movie time) were sequenced for each genome with a p-read yield of 39 Gb and a total coverage of ~ 110× per genome (Additional file 1: Table S11). The assembly was performed using FALCON-UNZIP [54], and the resulting sequence was polished using QUIVER [55]. Finally, to correct false polymorphisms resulting from errors in PacBio reads, homozygous SNPs and INDELs were corrected in the release consensus sequence using ~ 80× of the 2 × 250 Illumina reads from the reference individual. This was accomplished by aligning the reads using bwa mem and identifying homozygous SNPs and INDELs with the GATK’s UnifiedGenotyper tool [56] (Additional file 1: Table S12).

Chromosome-scale assemblies were created using a genetic map derived from 3697 GBS markers generated for a family of 497 F_2_ progeny from a cross in which the male reference is the father and the female reference is the grandmother. This map is described more completely in a previous publication [57]. This intercross map was used to identify misjoins, characterized by an abrupt change in the *S. purpurea* linkage group. Scaffolds were then oriented, ordered, joined, and numbered using the intercross map and the existing 94006 v1 release assembly [20]. Adjacent alternative haplotypes were identified on the joined contigs, and these regions were then collapsed using the longest common substring between the two haplotypes. Significant telomeric sequence was identified using the (TTTAGGG)_n_ repeat, and care was taken to make sure that it was properly oriented in the production assembly. The remaining scaffolds were screened against bacterial proteins, organelle sequences, and GenBank nr and removed if found to be a contaminant. Completeness of the euchromatic portion of the assembly was assessed by aligning *S. purpurea* var 94006 v1 annotated genes to the assemblies. In both cases, 99.7% of the genes were found.

### Identification of W contigs

Contigs derived from the W chromosome are expected to contain some large indels compared to contigs from the Z chromosome due to the lack of recombination between W and Z. These hemizygous regions should exclusively occur in the W haplotype of SDR. To identify these regions, we aligned 2 × 250 bp Illumina resequencing reads from female clone 94006 and male clone Fish Creek to the new reference using Bowtie2 [58]. Depth of coverage was extracted using samtools-1.2 [59]. Median depth was calculated using a non-overlapping sliding window of 10 kb.

To verify if these hemizygous regions are strictly inherited in only female individuals, we used the GBS data from the F_2_ family. GBS reads of 195 offspring of each sex were aligned to the v5 reference with Bowtie2. Due to low coverage and depth of the GBS markers per locus per individual, bam files were merged according to sex in samtools-1.2. Depth was then called in Samtools-1.2 with and max depth was limited to 80,000. Regions continuously covered by GBS reads were defined as GBS intervals. Then, the median of each sex was calculated across all of the intervals. We defined markers as female-specific by integrating the depth from both the F_2_ GBS and 2 × 250 datasets (restricted to the GBS intervals) using two rules: (1) log_2_($\frac{M_{195}+1}{F_{195}+1}$) < *L*, where *L* is the lower bounds of the distribution, defined by the fifth percentile divided by the number of intervals tested (Additional file 2: Figure S7), and (2) log_2_($\frac{94006_{2 by250}+1}{{\mathrm{Fish}\ \mathrm{Creek}}_{2 by250}+1}$) > 5. The cutoff for the second criterion was based on the occurrence of a distinct peak in the distribution of the ratios (Additional file 2: Figure S8). Scaffolds that contained at least three sex-linked markers were selected as candidate W scaffolds. Based on these criteria, only two contigs from the original Chr15 assembly were from W contigs, and the rest were from Z (Additional file 1: Table S5; Additional file 2: Figure S1a).

### Assembly of the Z and W chromosomes

Raw GBS reads used for the original map were demultiplexed and trimmed down to 64 bp for each read by process_radtags (in Stacks 1.44 [60]) with -c -q -r -t 64. Then, trimmed reads of each sequenced individual from the F_2_ family were aligned to the 19 chromosomes and unmapped scaffolds from the main genome and alternative haplotypes from the v4 reference of 94006 using Bowtie 2 [58] with the --very-sensitive flag (-D 20 -R 3 -N 0 -L 20 -i S,1,0.50) to maintain a balance between sensitivity and accuracy. Upon examining the distribution of SNPs in the genome, it became clear that the alternative haplotypes were preventing us from retrieving markers in some regions in the genome, so we repeated the alignments using three different reference sequences: (1) the 19 chromosomes, (2) unmapped scaffolds, and (3) alternative haplotypes. Then, a wrapper script ref_map.pl in Stacks was used to call genotypes with -m 5 (minimum number of reads to create a tag for parents) and -P 3 (minimum number of reads to create a tag for an offspring) on all progeny. Cross type “CP” was chosen since it was the one closest to our cross. Offspring with poor coverage were removed from the downstream analysis.

Once all genotypes were retrieved through Stacks, markers from different loci showing the exact same genotype/segregation across the progeny were binned and only markers from the main genome were kept for mapping. Markers with severe segregation distortion or excessive missing data were excluded, along with 12 offspring with a very low call rate. Genotypes were imputed and corrected based on inferring haplotypes in the two F_1_ parents from segregation of the markers in the progeny.

The grandparents of the F_2_ cross have extensive stretches of shared haplotypes, possibly due to historic inbreeding in this naturalized population. This results in long runs of heterozygosity and homozygosity in the F_1_ progeny. This inhibits integration of backcross and intercross markers by available mapping algorithms like those in the Onemap package [61]. To circumvent this problem, all intercross markers were translated to female and male backcross markers by identifying the parental origins of alleles based on parental phases and physical position in the assembly. Also, putatively hemizygous markers were recoded as backcross markers using sequence depth to infer genotypes. For example, markers with the segregation pattern +/− x −/− were recoded as AB x BB. These genotypes were also imputed and corrected based on the inferred haplotypes of the two F1 parents.

Onemap v2.1.1 was used to form initial linkage groups. For each chromosome, there are two phased linkage groups from each backcross type. However, this phase information derived from the F_2_ family is only for the F_1_ parents, which cannot be directly used for phasing haplotypes in the grandmother, clone 94006. By comparing parental genotypes from one LG to those of the grandparents, we inferred which of the 94006 haplotypes were inherited by each F_1_. These results were used as a piece of evidence for identifying W-linked scaffolds/contigs, as well as estimating the overall occurrence of chimeric contigs in the assembly. After building a framework genetic map using markers from the main genome, non-distorted markers from unmapped main scaffolds and alternative scaffolds were added.

All unmapped scaffolds were manually checked to see if they matched the phase information or contained sex-linked markers. Those that were identified as Z scaffolds/contigs were excluded from the W map. The new W and Z were assembled using the python package ALLMAPS [62] to order and orient scaffolds and reconstruct chromosomes based on the genetic map. Only the order of the female backcross map was used to assemble the W, and ALLMAPS was set not to break contigs. This new map-based assembly containing two versions of chromosome 15 (Chr15Z and Chr15W) is version 5 of the *S. purpurea* var 94006 genome.

To identify Z-W homologous regions (analogous to X-degenerate regions in mammalian sex chromosomes) and insertions in the W haplotype, we realigned the 2 × 250 reads of 94006 and Fish Creek to the 94006 v5 reference using Bowtie2 as described above, except we removed Chr15Z from the reference. Depth was calculated using samtools, and the median depth of 50-kb non-overlapping windows was calculated with an in-house perl script. Regions where medians of Fish Creek depth are no greater than 10 were considered as insertions in the FSW, and regions with greater depth were considered Z-W homologous regions. This analysis was repeated with a 10-kb window as well to enhance the resolution.

### Annotation of the genome

Transcript assemblies were constructed from ~ 126 M pairs of 2 × 76 bp (94006) or 2 × 150 bp (Fish Creek) paired-end Illumina RNA-seq reads using PERTRAN. A total of 188,628 transcript assemblies were constructed using PASA from the RNA-seq transcript assemblies. Loci were determined by transcript assembly alignments and/or EXONERATE alignments of proteins from *Arabidopsis thaliana*, soybean, poplar, cassava, brachypodium, grape, and Swiss-Prot proteomes, and high confidence *Salix purpurea* Fish Creek gene model peptides, with up to 2 kb extension on both ends unless extending into another locus on the same strand. The reference genome was soft-masked using RepeatMasker. Gene models were predicted by the homology-based predictors. FGENESH+, FGENESH_EST, and EXONERATE, by PASA assembly of ORFs, and from AUGUSTUS via BRAKER1. The best scored predictions for each locus were selected using multiple positive factors including EST and protein support, and one negative factor: overlap with repeats. The selected gene predictions were improved by PASA. Improvement included adding UTRs, splicing correction, and adding alternative transcripts. PASA-improved gene model proteins were subjected to protein homology analysis to the abovementioned proteomes to obtain Cscore (the ratio of mutual best hit BLASTP scores) and percentage of protein aligned to the best homolog. The transcripts were selected if its Cscore was greater than or equal to 0.5 and protein coverage greater than or equal to 0.5. Alternatively, proteins with EST coverage were accepted if overlap with repeats was less than 20%. For gene models with greater than 20% CDS overlap with repeats, the Cscore cutoff was 0.9 and homology coverage was at least 70%. The selected gene models were subjected to Pfam analysis, and gene models with more than 30% in Pfam TE domains were removed. Incomplete gene models with low homology and transcriptome support and short single exon proteins (< 300 BP CDS) lacking conserved domains or transcriptome support were manually filtered out.

To annotate potential genes or coding regions in the palindrome that were missed by the automated annotation, the full nucleotide sequence of arm1 (about 20 kb) was submitted to the Fgenesh online service (http://www.softberry.com/berry.phtml?topic=fgenesh) with specific gene-finding parameters for *Populus trichocarpa*. The predicted peptide sequences were searched against predicted proteins from *Populus trichocarpa* v3.0, and *Arabidopsis thaliana* TAIR10 in Phytozome 12 (https://phytozome.jgi.doe.gov/) to find the closest homologous annotation. The protein domains were identified using hmmscan in HMMER (v3.1b1, http://hmmer.org/) against the Pfam-A domains (release 32, https://pfam.xfam.org).

### Comparison of Z and W orthologous genes

Homologous genes on the Z and W chromosomes (Z-W homologs) were identified by performing a reciprocal blastp of all primary annotated peptide sequences in the main genome with default parameters. Mutual best hits were identified with over 90% identity over at least 70% of the transcript. Tandem duplications were identified as genes with expectation values of 1 × 10^− 10^ that occurred within a 500-kb window. In these cases, one representative gene from each tandem array was used as a representative sequence, and the mutual best hit outside the tandem array was identified as above. Genes that lacked hits in the Z-SDR were searched against the *Populus trichocarpa* v3.0 reference genome. Those with hits to Chr15 in Populus were designated as “Ancestral” under the assumption that the homolog was present prior to the establishment of the SDR in *S. purpurea*, but was subsequently lost from the Z-SDR. Those genes that lacked hits to Chr15 in either species but which had a mutual best hit meeting the above criteria to an autosomal gene were designated as autosomal transpositions. Genes that could not be readily categorized due to a lack of mutual best hits satisfying the above criteria were designated as “Non-mutual” or “No Hit” as appropriate.

To identify homologous gene pairs for calculation of synonymous substitutions between the Z and W alleles, a reciprocal blast of all primary annotated peptide sequences was run with “blastall –p blastp -i -e 1e-20 -b 5 -v 5 -m 8”, and MCscanX was run with default parameters [63]. The synonymous and nonsynonymous substitution rate of each gene pair in each syntenic block (*d*_S_ and *d*_N_, respectively) was estimated by aligning the sequences with CLUSTALW [64] and using the yn00 function in PAML [65]. Only pairs between the W-SDR and Z-SDR (including the unmapped scaffold_844) were used for estimating the divergence between Z and W haplotypes. It is important to note that this analysis does not control for polymorphism within populations, so it may be an overestimate of divergence.

### Identification of sex-associated loci

Loci associated with sex were identified using 60 non-clonal individuals from a naturalized population of *S. purpurea* [66]. GBS reads from each individual were aligned to the 94006v5 genome without Chr15Z using Bowtie2. Genotypes were called in Stacks 1.14 using the ref_map.pl wrapper and the population module with a minimum minor allele frequency of 0.1 and a genotyping rate of 0.1. Loci with greater than 40% missing data were removed. Association with sex was performed using emmax [67] as described previously [20].

### Detection of palindromic repeats

We detected the palindromic repeats by aligning the SDR region to itself with LASTZ 1.03.66 with the following flags: --gapped --exact = 100 --step = 20. Paralogous gene copies on autosomes were retrieved from the reciprocal blastp results described above. Paralogous genes within the palindrome arms were aligned along with paralogous copies from the autosomes using MUSCLE using default parameters provided in MEGA 5. In a few cases, the resulting alignments were adjusted manually (Supplemental Materials: Additional file 3). A Neighbor-Joining tree with default parameters was built using MEGA 5 [68].

To identify recent insertions of transposable elements within the palindrome, LTRharvest [69] was run with the sequence of the palindromic portion of the W-SDR from 8778 to 9015 kb with the target site duplication restricted to 5 to 20 bp. To find the protein domains in the coding region, a protein domain search against Pfam-A domains (release 32) was performed using the hidden Markov model methods implemented in LTRdigest (–hmms flag) [70]. Predicted LTR retrotransposons were determined to be non-automonous when coding regions did not contain any *gag*- or *pol*-related domains.

We estimated time since transposition based on the number of substitutions between the two LTR arms [71]. To estimate the substitution rate between the flanking LTR repeats, 5′ and 3′ repeats of each LTR retrotransposon predicted from LTRharvest were aligned by MUSCLE using default parameters provided in MEGA 5. After all gaps were removed, both number of differences and substitution rate were estimated in MEGA5. For number of differences, transitions and transversions were both included with a uniform rate. Substitution rate was modeled using the Kimura 2-parameter model provided in MEGA5, and the rate variation among sites was modeled with a gamma distribution (shape parameter = 1). The time since transposition was estimated based on the mutation rate previously reported for *Populus tremula* (2.5 × 10^− 9^ per year) [29].

### Detection of gene conversion

As evidence of gene conversion, we searched for regions that were differentiated between species but concordant among the palindrome arms [31]. To accomplish this, we first aligned paired-end reads from a female clone of *S. suchowensis* (srx1561933) to the 94006 v5 female reference, plus alternative haplotypes, using Bowtie2 with the --local flag. This yielded an 82.9% overall alignment rate on average. The Illumina reads described above for clone 94006 were mapped using identical parameters. All reads aligning to the palindromes were extracted and compared to the whole genome using blastn. Mis-mapped reads originating from the autosomes were manually identified by scrutinizing the alignments, and only reads that mapped exclusively to the palindromic regions were retained. These reads were then re-aligned to a new reference consisting exclusively of arm 1 of the *S. purpurea* palindrome. SNPs and indels were called using mpileup and filtered to exclude loci with a minimum site quality <Q20 or depth > 300.

### Expression profiling

RNAseq data was obtained from catkins of 10 female and 10 male F_2_ progeny. RNAseq data were also obtained from multiple tissues of clones 94006 and Fish Creek. All sequences were Illumina 2 × 150 bp reads, except for 94006, which were 2 × 76 bp reads. Transcripts from the palindrome can have high sequence identity among arms and with other paralogous sequences on the autosomes, which can complicate estimation of gene expression. Thus, all predicted coding sequences from the same gene family in the palindrome were aligned to the autosomal paralogs, and conserved sequences were masked in the reference genome. Salmon-0.11.3 [72] was used to quantify (salmon quant) the raw read count for each sample mentioned above with the gcBias flag as suggested by the developers. Heatmaps were generated separately for each group of palindrome genes, using log_2_ transformed data normalized with respect to library size or by variance stabilizing transformations (VST) using the R packages pheatmap and Deseq2 [73].

## Supplementary information

Additional file 1 Supplementary tables.

Additional file 2 Supplementary figures.

Additional file 3 Review history.
