# Supplementary material for: A willow sex chromosome reveals convergent evolution of complex palindromic repeats
Source: Genome Biol. 2020 Feb 14;21:38. doi: 10.1186/s13059-020-1952-4 (PMC7023750; doi:10.1186/s13059-020-1952-4)
Supplement: Supplementary file 3 — Review history. [file 13059_2020_1952_MOESM3_ESM.docx]

Review History

**First round of review**

**Reviewer 1**

**Comments to author:**

Sex chromosome palindromes consist of inverted duplicates that allow for local recombination in an otherwise nonrecombining chromosome. The theory of this was beautifully discussed by E. Betrán, J.P. Demuth, and A. Williford in their paper "Why chromosome palindromes? Int. J. Evol. Biol. 2012; 2012: 207958, which Zhou and colleagues might want to read and cite because they here report the first clear case of palindromes on plant sex chromosomes. On the Salix pupurea W chromosome, they found two consecutive palindromes spanning over a region of 200 kb, with conspicuous 20 kb stretches of highly conserved sequences among the four arms. In the closely-related species S. suchowensis they found two copies of a portion of the same palindrome, providing evidence for gene conversion. Four genes in the palindrome are homologous to genes in the Sex Determining Region (SRD) of the Salix sister genus Populus, which is located on a different chromosome than the Salix SRD. After the palindromes were established, several retrotransposons inserted themselves into the palindromic genes. Unfortunately, there is still no definitive order for the female-specific contigs due to lack of recombination in the SDR.

These findings are important, and the paper is clear and well-structured. To my mind, the two pages in the Discussion about the potential role of cytokinin signaling in dioecy go too far / could be shortened; this is all only based on the BLAST homology of the multi-copy genes in the palindromes to type A cytokinin response regulator homologs also found in Arabidopsis.

**Reviewer 2**

**Comments to author:**

I found this ms very interesting. It describes what appears to be an impressive analysis of the genome sequence of a Salix species, S. purpurea. It had previously been established that this dioecious plant has a genetic sex-determining system, and that, as in other species in this genus, females are the heterogametic sex. Now, building on previous work by this group, extensive W- and Z-linked regions have been identified on chromosome 15. The former apparently contain "palindrome" sequences that partly explain its larger number of genes, and also perhaps carry genes that are also found in the chromosome 19 sex-determining of Populus species, despite the fact that these have male heterogamety. However, low mapping quality cutoffs were used, and may be inappropriate for a large genome. Steps do not seem to have been taken to ensure that unique reads are mainly being examined, despite this choice, so this may exaggerate signals in repetitive regions, and the findings of palindromes might be affected.


I don't see the need for the Conclusions section, as it adds nothing to what has already been explained perfectly clearly.

However, I have some concerns. The pages are not numbered, so I will refer to line numbers.

1.     One issue is that the ms mentions Y chromosome palindromes, but not X chromosome ones.

2.     I did not understand the brief mention of "sex association" in line 90. It appears that only a single male and female were sequenced, and so any association analysis is weak. Of course, a different depth of coverage in the genome sequence data from one of each sex can reliably suggest the existence of a fully sex-linked region, if it is physically extensive enough. The text mentions validating sex-linkage using markers whose design is passed over much too briefly in line 96 — the marker design approach should be explained. Very large numbers of markers were mapped, but it is not clear enough how many were in the sex-linked region. Line 107 mentions "sex associated SNPs", and this concept needs at least a brief explanation.

Similarly, I did not understand the "associated with sex" remark in line 395. Do you mean fully sex-linked? If so, why not say that. If not, please can this be revised to become clearer.

3.     Line 28: "Sex chromosomes […] contain […] master regulatory genes that confer sex-specific traits". This is not necessarily true, although clearly there is probably a major sex-determining factor. It would therefore be best to avoid the language of 'master regulators' in general.

4.     Another mystifying concept is "Z degenerate genes" (line 144). I expected genetic degeneration to be detected in terms of Z-linked genes being absent from the Z, consistent with the authors' use of lower coverage in females as a way to identify the fully sex-linked regions.

5.     In relation to this part of the ms, it is good to see estimated synonymous site divergence (Ks) values between the W and Z genes, for the 146 single copy best hit genes from the fully sex-linked region, but the figure ought to show confidence intervals (and the mention of this estimate in line 315 should mention that it is Z-W divergence, presumably "raw" divergence, not correcting for sequence variation among the Z copies, or the W copies if more than a single W allele was included for each gene). The overall mean value of only 2.6% is interesting, given that S. trichocarpa has a sex-determining locus in apparently the same genome region (if I understand correctly — this is not completely clear from the text), and is much more diverged than the divergence between these 2 species (for some reason, this species' divergence value is not mentioned in line 313 onwards). Perhaps only a younger stratum has been found? Is there any evidence bearing on the possibility that the fully sex-linked region identified in this species might not actually include the sex-determining locus itself. An older, highly degenerated, stratum carrying few genes would be hard to detect, but should not be ruled out, and therefore it might merit a mention.

6.     Line 141 onwards mentions that the W-linked region has more genes than the Z-linked region (488 versus 317), but the reason for the difference is not explained. Although this might be partly explained by the presence of palindrome sequences in the W-linked region, it seems too large a difference for this to be true. The ms does not make clear whether both the regions are complete, so maybe the reason for the difference is simply that part of the Z-linked region is missing? It would seem odd that this would occur, as the ZZ male genome should be better assembled than the ZW female one, and I think this should be discussed briefly, as it relates to the trustworthiness of the assembly in general (which is, of course, discussed in the text, with appropriate caveats concerning difficulties). Another point regarding the gene number is that it would be helpful to state explicitly that the genes counted are plant genes, after excluding genes in the apparently abundant transposable elements (presumably, those were indeed excluded).

7.     Some parts of the Methods need to be justified.

a.     Line 459: Why was a quality < 20 chosen, rather than a more typical value of quality < 30 (see: http://maq.sourceforge.net/qual.shtml#mapQ2)? A mapping quality of 20 is a threshold where 1 in 100 reads will map incorrectly. Whilst not all of their data will be at such a quality (as many will have MQ over 30), this is not good practice, especially with such a large data set of many millions of reads, allowing a more stringent cutoff than is usual. This is especially important for finding palindromic regions, which are notoriously difficult to map reads to, because they can resemble repetitive elements, transposable elements and other contamination that may be present in reads (though the authors have sought to address these well). Perhaps the authors can confirm that their reads between MQ 20-30 are robust (mainly uniquely mapping)?
b.     Line 484: bwa mem is used for alignment to detect SNPs and indels (line 464) but Bowtie2 for aligning reads when examining coverage differences in males vs females, to produce W contigs (line 487). This makes it harder to compare findings, as bwa mem is likely to produce a much greater mapping (meaning the recovered SNPs are more representative, whereas the recovered W contigs are conservative). See: https://www.biorxiv.org/content/biorxiv/early/2016/05/16/053686.full.pdf especially Figure 4. This may have been intended, however it may have also resulted in an overly-strict finding of fewer W contigs, given the criterion of peak detection used (line 499). It should certainly be explained.

c.     The SNP distribution (bwa mem-based) and a stringent Bowtie2-based alignment of F2 family reads are seemingly interpreted together (in line 511) without a comment that the different alignment methods might be impacting the result of attempting alignment to retrieve markers in certain regions (whereas these markers might have been retrieved if a bwa mem-based approach was adopted throughout, as bwa mem was used in production of the haplotypes). Whilst they work around this with a creative method (line 513), this may also indicate that mapping in these supposed haplotype regions is fluid relative to alignment method used, decreasing confidence in the validity of the haplotypes. This may have been intended in order to use results from multiple tools to improve confidence, but that does not seem to be the authors intent. Again, an explanation would be helpful.


Minor comments (some not so minor)
1.     It is thought that Y chromosome palindromes facilitate sequence conservation through gene conversion, but it is too strong to say simply that they do so. The existence of X chromosome palindromes cannot be explained in this way. Given this, I feel that the long Discussion section paragraph about this possibility should be shortened, to make the point that it is one possibility.
2.     It is rather misleading to say that "Plant sex chromosomes have been identified in approximately 40 species, but fewer than half of them are heteromorphic". The term "sex chromosome" is often used to indicate that there is a heteromorphic pair carrying the sex-determining locus, so this sentence could be confusing. The 40 species mentioned are plants thought to have genetic systems that were mostly studied by cytogenetic means quite long ago, and many other dioecious plants have not been studied, so the number represents the species studied, only a minority of dioecious angiosperms. No heteromorphism was found in about half of them, and this could be be representative of angiosperms in general. The non-heteromorphic species in these studied might well have heteromorphism that could be detected by genome sequencing and assembly, so the reviews cited are really reporting only quite old (and pretty uncertain) information.
3.     The phrase "highly identical" should be changed to identical (if that is the meaning) or to "highly similar".
4.     The best evidence of the presence of Y-linked female-suppressing factors is in Silene, as reviewed in Westergaard's 1958 paper, which should be cited near lien 45 (The mechanism of sex determination in dioecious plants. Advances in Genetics, 9, 217-281). However, the sentences about RNA-seq at the end of the paragraph seem irrelevant to the topic of the ms.
5.     The last paragraph of the Introduction is clumsily written, and I think means the following (although I did not understand the meaning of female-biased alleles, which appears in several places in the ms — if you mean female-specific variants, this term should be used — nor did I understand "a large number of female-biased alleles" —if these sequences have many different alleles, some explanation should be offered).

Sex determination is similarly diverse within the Salicaceae family. SDRs with female heterogamety have been found on chromosome 15 in multiple Salix species [18-
20], whereas most species in the closely-related genus Populus show male heterogamety and their sex determining regions map to chromosome 19 [21,22]. We previously reported that the S. purpurea SDR occupies a large portion of the W chromosome (Chr15) that does not recombine, and in which sequences have a large number of female-biased alleles [18,23], whereas small SDRs are inferred in P. trichocarpa and P. balsamifera [22,24]. Here we present a much more complete assembly of the S. purpurea W-linked region and report, for the first time in plants, a palindromic repeat structure that is similar to that of mammalian Y chromosomes. We also demonstrate that genes are shared by the Salix and Populus SDRs, suggesting that they may share sex determination mechanisms in the two genera in this family, despite having different heterogamety, and that the different genomic locations may reflect transpositions of the sex determining locus.

6.     An important issue is the use of the term "sex-biased". This conveys the idea of a larger quantity of some sequence in one sex than the other, and the term is widely used in studying gene expression, where it seems appropriate. However, line 87 says "sex-biased markers", and it is not clear if this is simply a typo for "sex-linked", or whether something else is meant. This usage appears in several places (e.g. Line 56) and should not be used if the meaning is "sex-linked. It is also ok to say "sex-specific depth", as in line 123, but I think that it would be preferable to be more explicit, e.g. "low depth of coverage of the inferred fully sex-linked region in females".
7.     Line 88 would be clearer as "W scaffolds in a region we infer to be within the SDR….".
8.     In mentioning gene density values of the W- and Z-linked regions (line 133 onwards), it would be helpful to mention that the lower values than for autosomal regions suggests that repetitive sequences have accumulated.
9.     The phrase in line 308 "linkage between autosomal and sex linked genes" is odd, and maybe means "linkage between sex linked and formerly autosomal genes"
10.     When sexual antagonism is mentioned, e.g. in line 323, it ought to be made clear that it is sexually antagonistic polymorphisms that are important, and in line 330 it would be better to say "multi-step" rather than "gradual" when referring to cessation of recombination.
11.     The text refers in line 334 to "small" the fully sex-linked sex-determining regions of Populus species, but it would be good to be more clear and specific about sizes.
12.     Line 355 mentions two "major autosomal translocations", but no details or references are given.
13.     When mentioning Arabidopsis thaliana, it whould be given its full name the first time it appears.

**Authors Response**

**Point-by-point responses to the reviewers’ comments:**

Reviewer #1

Sex chromosome palindromes consist of inverted duplicates that allow for local recombination in an otherwise nonrecombining chromosome. The theory of this was beautifully discussed by E. Betrán, J.P. Demuth, and A. Williford in their paper "Why chromosome palindromes? Int. J. Evol. Biol. 2012; 2012: 207958, which Zhou and colleagues might want to read and cite because they here report the first clear case of palindromes on plant sex chromosomes. *Response*

*We thank the reviewer for pointing out this excellent review. We have cited it in the Introduction and Discussion of the revised manuscript.*

On the Salix pupurea W chromosome, they found two consecutive palindromes spanning over a region of 200 kb, with conspicuous 20 kb stretches of highly conserved sequences among the four arms. In the closelyrelated species S. suchowensis they found two copies of a portion of the same palindrome, providing evidence for gene conversion. Four genes in the palindrome are homologous to genes in the Sex Determining Region (SRD) of the Salix sister genus Populus, which is located on a different chromosome than the Salix SRD.

After the palindromes were established, several retrotransposons inserted themselves into the palindromic genes. Unfortunately, there is still no definitive order for the female-specific contigs due to lack of recombination in the SDR.

These findings are important, and the paper is clear and well-structured. To my mind, the two pages in the Discussion about the potential role of cytokinin signaling in dioecy go too far / could be shortened; this is all only based on the BLAST homology of the multi-copy genes in the palindromes to type A cytokinin response regulator homologs also found in Arabidopsis.

*We have shortened the portion of the discussion that describes the cytokinin signaling pathway and its possible role in sex determination.*

Reviewer #2

I found this ms very interesting. It describes what appears to be an impressive analysis of the genome sequence of a Salix species, S. purpurea. It had previously been established that this dioecious plant has a genetic sex-determining system, and that, as in other species in this genus, females are the heterogametic sex. Now, building on previous work by this group, extensive W- and Z-linked regions have been identified on chromosome 15. The former apparently contain "palindrome" sequences that partly explain its larger number of genes, and also perhaps carry genes that are also found in the chromosome 19 sex-determining of Populus species, despite the fact that these have male heterogamety. However, low mapping quality cutoffs were used, and may be inappropriate for a large genome. Steps do not seem to have been taken to ensure that unique reads are mainly being examined, despite this choice, so this may exaggerate signals in repetitive regions, and the findings of palindromes might be affected.

*We believe that the reviewer is referring to our evaluation of gene conversion, described in lines 649 to 657 of the submitted manuscript. Upon revisiting the methods used for this portion of the analysis, we discovered that the quality cutoff was for site quality for the individual SNP calls, and not mapping quality, as originally stated in the manuscript. This has been corrected in the revision, and we apologize for the confusion.*

*Nevertheless, the robustness of this analysis is an essential component of the manuscript, so it merits further explanation here. The purpose of this analysis was to detect duplicated copies of the palindrome arms in Salix suchowensis that might have been undergoing gene conversion, as we observed for the closely-related S. purpurea. The palindromic portion of the SDR region was not present in the publicly available S. suchowensis genome assembly which was based on short sequence reads (454, and Illumina sequence). We were therefore forced to use reference-based alignments of Illumina sequence reads to evaluate gene conversion in S. suchowensis. The portion of the analysis that used a Q20 cutoff was not based on alignments to the entire S. purpurea genome. Instead the reference used for SNP identification was ONLY arm1 of the palindrome. The purpose was to detect all matching reads in the S. suchowensis genome, especially including those reads resulting from duplicated loci such as those present in a palindrome undergoing gene conversion. Therefore the use of relatively low base quality cutoffs was conservative because a LACK of intraspecific polymorphisms is the primary evidence for gene conversion. The fact that we detected duplicated loci (based on relative sequence depth) that had high conservation within S. suchowensis and S. purpurea, but high divergence between the two species, is strong evidence for gene conversion, and this conclusion would not be affected by a higher site quality cutoff.*

*We have clarified this portion of the methods to better explain the rationale for the two different alignments (whole genome reference vs Arm1 reference) (lines 644 to 656).*

I don't see the need for the Conclusions section, as it adds nothing to what has already been explained perfectly clearly.

*The Genome Biology guidelines specify that research articles should contain a Conclusions section that provides “a brief summary and potential implications.” https://genomebiology.biomedcentral.com/submissionguidelines/preparing-your-manuscript/research*

*We have added an additional sentence to the Conclusions to point out one more potential implication of our work “If the underlying mechanism is the same, this will be the first case demonstrating XY and ZW systems that are controlled by the same pathway in plants.”*

However, I have some concerns. The pages are not numbered, so I will refer to line numbers.

*We have added page numbers to the revised manuscript.*

1. One issue is that the ms mentions Y chromosome palindromes, but not X chromosome ones.

*We revised the second paragraph of the introduction to acknowledge that both X and Y chromosomes contain palindromes in mammals, and also that palindromes occur in other parts of the genome as well, but are most prevalent on the sex chromosomes.*

2. I did not understand the brief mention of "sex association" in line 90. It appears that only a single male and female were sequenced, and so any association analysis is weak. Of course, a different depth of coverage in the genome sequence data from one of each sex can reliably suggest the existence of a fully sex-linked region, if it is physically extensive enough. The text mentions validating sex-linkage using markers whose design is passed over much too briefly in line 96 — the marker design approach should be explained.

*We used two complementary approaches for identifying sex-specific contigs. One relied upon association of SNP alleles with sex in a population of 60 unrelated individuals from a naturalized population. The second method was based on depth of GBS markers in 195 male and 195 female full sibs from an F2 family, complemented by deep whole genome resequencing data from a single male and a single female individual. This is explained in the Methods section, but since that comes at the end of the manuscript, we have briefly summarized those methods within the Results section to clarify our approach.*

Very large numbers of markers were mapped, but it is not clear enough how many were in the sex-linked region. Line 107 mentions "sex associated SNPs", and this concept needs at least a brief explanation. Similarly, I did not understand the "associated with sex" remark in line 395. Do you mean fully sex-linked? If so, why not say that. If not, please can this be revised to become clearer.

*For this portion of the manuscript we are using the phrase “sexassociated markers” because this best describes the results of the GWAS analysis for sex, which is explained more fully in the Methods section. We have clarified this section by specifying “for the 60 unrelated individuals.” We have also changed “sex-associated” to “sexlinked” for further clarification.*

3. Line 28: "Sex chromosomes […] contain […] master regulatory genes that confer sex-specific traits". This is not necessarily true, although clearly there is probably a major sex-determining factor. It would therefore be best to avoid the language of 'master regulators' in general.

*We have removed this phrase.*

4. Another mystifying concept is "Z degenerate genes" (line 144). I expected genetic degeneration to be detected in terms of Z-linked genes being absent from the Z, consistent with the authors' use of lower coverage in females as a way to identify the fully sex-linked regions.

*We adopted this phrase directly from the mammalian literature (Xdegenerate genes). We added some text to clarify that Z-degenerate refers to regions that are present on both the Z and W chromosomes.*

5. In relation to this part of the ms, it is good to see estimated synonymous site divergence (Ks) values between the W and Z genes, for the 146 single copy best hit genes from the fully sex-linked region, but the figure ought to show confidence intervals.

*We have added a new Figure 3 with confidence intervals. We changed Ks to dS to facilitate the calculation of the confidence intervals. This did not change our conclusions in any way.*

(and the mention of this estimate in line 315 should mention that it is Z-W divergence, presumably "raw" divergence, not correcting for sequence variation among the Z copies, or the W copies if more than a single W allele was included for each gene).

*The comparison of the Z and W gene copies was based on the single phased copies that were present in the sequenced individual. Intraspecific variation in these loci would therefore not be reflected in these values. We have clarified the methods to make this more apparent. We have also noted that this is raw divergence in the Methods (lines 611- 613).*

The overall mean value of only 2.6% is interesting, given that S. trichocarpa has a sex-determining locus in apparently the same genome region (if I understand correctly — this is not completely clear from the text), and is much more diverged than the divergence between these 2 species (for some reason, this species' divergence value is not mentioned in line 313 onwards). Perhaps only a younger stratum has been found?

*The SDR of P. trichocarpa occurs on a different chromosome (Chr19) and is an XY system. This is stated on line 62 of the original submission. From this standpoint, it makes sense that the SDR would be relatively young in both species. We have now noted the location of the P. trichocarpa SDR in the revised version of the discussion (line 333). This manuscript does not describe divergence between X and Y copies of genes in the Populus SDR. That work is from a prior manuscript from another group (ref 25 in the revised manuscript).*

*If the reviewer is referring to S. suchowensis, which does have an SDR in approximately the same region, then the results still make sense, because the divergence between S. purpurea and S. suchowensis is about the same as the Z-W divergence observed within S. purpurea. This is shown in Fig. 3b. We do not think there are any inconsistencies in these results.*

Is there any evidence bearing on the possibility that the fully sex-linked region identified in this species might not actually include the sex-determining locus itself. An older, highly degenerated, stratum carrying few genes would be hard to detect, but should not be ruled out, and therefore it might merit a mention.

*Yes, this is an excellent point. We have acknowledged this possibility in the revised Discussion: “It appears instead that cessation of recombination has not been a gradual long-term process in the S. purpurea SDR, although it is certainly possible that the oldest strata have decayed to the point where they cannot be meaningfully aligned.”*

6. Line 141 onwards mentions that the W-linked region has more genes than the Z-linked region (488 versus 317), but the reason for the difference is not explained. Although this might be partly explained by the presence of palindrome sequences in the W-linked region, it seems too large a difference for this to be true. The ms does not make clear whether both the regions are complete, so maybe the reason for the difference is simply that part of the Zlinked region is missing? It would seem odd that this would occur, as the ZZ male genome should be better assembled than the ZW female one, and I think this should be discussed briefly, as it relates to the trustworthiness of the assembly in general (which is, of course, discussed in the text, with appropriate caveats concerning difficulties).

*This was an excellent suggestion, and we have now included additional text in the Results section that speaks to the completeness of the female Z assembly: “To evaluate the completeness of the Z chromosome, we compared the gene content of this region to that from the Fish Creek male reference genome. The Z-SDR region was comprised of four contigs spanning from 2.86 to 7.10 Mb in Fish Creek, containing a total of 333 genes. Since the size and gene content was very similar between the Z chromosomes of the male and female references, we are restricting our analysis to the female to simplify the comparison.”*

*Regarding the source of differences in gene content in the W and Z SDR regions, Figure 2 shows the abundance of genes in different categories on the W and Z chromosomes. We point out that a large source of the discrepancy is translocation of autosomal genes to the WSDR. This accounts for about half of the genes that are present on the W but absent on the Z. All categories were represented by more genes on the W, with the exception of tandem-duplications of Z-degenerate genes (i.e., those genes that had mutual best hits on both chromosomes).*

Another point regarding the gene number is that it would be helpful to state explicitly that the genes counted are plant genes, after excluding genes in the apparently abundant transposable elements (presumably, those were indeed excluded).

*Yes, genes derived from transposable elements were filtered out, as described in the Annotation of the genome section of the Methods: “The selected gene models were subjected to Pfam analysis and gene models with more than 30% in Pfam TE domains were removed.” We have revised the entire section to improve clarity.*

7. Some parts of the Methods need to be justified.

a. Line 459: Why was a quality < 20 chosen, rather than a more typical value of quality < 30 (see: http://maq.sourceforge.net/qual.shtml#mapQ2)? A mapping quality of 20 is a threshold where 1 in 100 reads will map incorrectly. Whilst not all of their data will be at such a quality (as many will have MQ over 30), this is not good practice, especially with such a large data set of many millions of reads, allowing a more stringent cutoff than is usual. This is especially important for finding palindromic regions, which are notoriously difficult to map reads to, because they can resemble repetitive elements, transposable elements and other contamination that may be present in reads (though the authors have sought to address these well). Perhaps the authors can confirm that their reads between MQ 20-30 are robust (mainly uniquely mapping)?

*We certainly agree with the reviewer that comparing polymorphisms discovered using different alignment algorithms would be a serious problem. However, all comparisons in this manuscript are based on our Bowtie2 alignments. The bwa mem alignments described in the genome assembly were only used during the assembly process. Furthermore, the Q20 quality cutoff does not refer to mapping quality, but instead refers to the base quality criterion used for read trimming prior to alignment. Furthermore, the bwa mem alignments using the trimmed reads were solely for the purpose of correcting base call errors and indels that were introduced during the PacBio sequencing and assembly process. Only polymorphisms that appeared to be homozygous based on aligning reads from the reference individual were corrected, as is a standard practice for PacBio assemblies. We have edited that section of the methods to make this more clear (lines 459-472).*

b. Line 484: bwa mem is used for alignment to detect SNPs and indels (line 464) but Bowtie2 for aligning reads when examining coverage differences in males vs females, to produce W contigs (line 487). This makes it harder to compare findings, as bwa mem is likely to produce a much greater mapping (meaning the recovered SNPs are more representative, whereas the recovered W contigs are conservative). See: <https://www.biorxiv.org/content/biorxiv/early/2016/05/16/053686.full.pdf> especially Figure 4. This may have been intended, however it may have also resulted in an overly-strict finding of fewer W contigs, given the criterion of peak detection used (line 499). It should certainly be explained.

*Please see the response to point 7a. SNPs were called from Bowtie2 alignments for the purpose of performing mapping and sex association analyses. This is explained in the Methods section Identification of W contigs.*

c. The SNP distribution (bwa mem-based) and a stringent Bowtie2-based alignment of F2 family reads are seemingly interpreted together (in line 511) without a comment that the different alignment methods might be impacting the result of attempting alignment to retrieve markers in certain regions (whereas these markers might have been retrieved if a bwa mem-based approach was adopted throughout, as bwa mem was used in production of the haplotypes). Whilst they work around this with a creative method (line 513), this may also indicate that mapping in these supposed haplotype regions is fluid relative to alignment method used, decreasing confidence in the validity of the haplotypes. This may have been intended in order to use results from multiple tools to improve confidence, but that does not seem to be the authors intent. Again, an explanation would be helpful.

*While the reviewer was mistaken about our use of bwa mem for SNP identification, we do agree that the algorithms underlying the different alignment programs could have an impact on our detection of W and Z contigs. We therefore repeated the analyses using alignments based on bwa mem. Although this resulted in identification of a larger number of unfiltered polymorphisms than the bowtie2 alignments, a subsequent test of sex association for these markers did not result in identification of new sex-linked contigs. We therefore believe that our results are robust to the choice of alignment program. We thank the reviewer for pointing out this possible source of artifacts.*

Minor comments (some not so minor)

1. It is thought that Y chromosome palindromes facilitate sequence conservation through gene conversion, but it is too strong to say simply that they do so. The existence of X chromosome palindromes cannot be explained in this way. Given this, I feel that the long Discussion section paragraph about this possibility should be shortened, to make the point that it is one possibility.

*We thank the reviewer for this insight. We have modified the discussion to moderate our assertion that palindromes provide an essential function in maintaining coding sequence integrity in the absence of recombination. We have also acknowledged other explanations for the roles of palindromes in sex chromosomes: “However, it is also important to note that not all palindromic repeats occur in regions of the genome with suppressed recombination, most notably the large palindromes on the mammalian X chromosome. Palindromes may therefore play another role beyond maintenance of sequence integrity, such as mitigating expression of sexually antagonistic genes [10] or in gene dosage compensation in the heterogametic sex [38,39].”*

2. It is rather misleading to say that "Plant sex chromosomes have been identified in approximately 40 species, but fewer than half of them are heteromorphic". The term "sex chromosome" is often used to indicate that there is a heteromorphic pair carrying the sex-determining locus, so this sentence could be confusing. The 40 species mentioned are plants thought to have genetic systems that were mostly studied by cytogenetic means quite long ago, and many other dioecious plants have not been studied, so the number represents the species studied, only a minority of dioecious angiosperms. No heteromorphism was found in about half of them, and this could be be representative of angiosperms in general. The non-heteromorphic species in these studied might well have heteromorphism that could be detected by genome sequencing and assembly, so the reviews cited are really reporting only quite old (and pretty uncertain) information.

*We have reworded the sentence to more accurately reflect the state of knowledge, and have cited a more definitive and appropriate review (Bachtrog et al., 2014): “A striking feature of plant sex chromosomes is a lack of obvious heteromorphism in most dioecious species (i.e., differences are not readily discernable by cytology) [12,13].”*

3. The phrase "highly identical" should be changed to identical (if that is the meaning) or to "highly similar".

*We have changed this to “highly similar” in some places and to “high sequence identity” in others. This is a broadly recognized term used to refer to DNA alignments.*

4. The best evidence of the presence of Y-linked female-suppressing factors is in Silene, as reviewed in Westergaard's 1958 paper, which should be cited near lien 45 (The mechanism of sex determination in dioecious plants. Advances in Genetics, 9, 217-281). However, the sentences about RNA-seq at the end of the paragraph seem irrelevant to the topic of the ms.

*We have rewritten that portion of the Introduction as follows: “The genus Silene has been a long-standing model for sex determination in XY plants, and female-suppressing and male-promoting factors were identified in the 1950’s using genetic approaches [19]. More recently it has been shown that some species of Silene have ZW sex determination systems, though it remains unclear if the underlying mechanism of sex determination in XY and ZW species is the same [20].”*

5. The last paragraph of the Introduction is clumsily written, and I think means the following (although I did not understand the meaning of femalebiased alleles, which appears in several places in the ms — if you mean female-specific variants, this term should be used — nor did I understand "a large number of female-biased alleles" —if these sequences have many different alleles, some explanation should be offered).

*We have revised the paragraph to make it clearer and more specific by including information about the estimated sizes of the SDRs in each species. We removed the reference to female-biased alleles, which detracted from the clarity of the point we were trying to make.*

Sex determination is similarly diverse within the Salicaceae family. SDRs with female heterogamety have been found on chromosome 15 in multiple Salix species [18- 20], whereas most species in the closely-related genus Populus show male heterogamety and their sex determining regions map to chromosome 19 [21,22]. We previously reported that the S. purpurea SDR occupies a large portion of the W chromosome (Chr15) that does not recombine, and in which sequences have a large number of female-biased alleles [18,23], whereas small SDRs are inferred in P. trichocarpa and P. balsamifera [22,24]. Here we present a much more complete assembly of the S. purpurea W-linked region and report, for the first time in plants, a palindromic repeat structure that is similar to that of mammalian Y chromosomes. We also demonstrate that genes are shared by the Salix and Populus SDRs, suggesting that they may share sex determination mechanisms in the two genera in this family, despite having different heterogamety, and that the different genomic locations may reflect transpositions of the sex determining locus.

6. An important issue is the use of the term "sex-biased". This conveys the idea of a larger quantity of some sequence in one sex than the other, and the term is widely used in studying gene expression, where it seems appropriate. However, line 87 says "sex-biased markers", and it is not clear if this is simply a typo for "sex-linked", or whether something else is meant. This usage appears in several places (e.g. Line 56) and should not be used if the meaning is "sex-linked. It is also ok to say "sex-specific depth", as in line 123, but I think that it would be preferable to be more explicit, e.g. "low depth of coverage of the inferred fully sex-linked region in females".

*We have changed sex-biased to sex-linked throughout the manuscript.*

7. Line 88 would be clearer as "W scaffolds in a region we infer to be within the SDR….".

*Fixed.*

8. In mentioning gene density values of the W- and Z-linked regions (line 133 onwards), it would be helpful to mention that the lower values than for autosomal regions suggests that repetitive sequences have accumulated.

*Done.*

9. The phrase in line 308 "linkage between autosomal and sex linked genes" is odd, and maybe means "linkage between sex linked and formerly autosomal genes"

*Fixed.*

10. When sexual antagonism is mentioned, e.g. in line 323, it ought to be made clear that it is sexually antagonistic polymorphisms that are important, and in line 330 it would be better to say "multi-step" rather than "gradual" when referring to cessation of recombination.

*Fixed.*

11. The text refers in line 334 to "small" the fully sex-linked sex-determining regions of Populus species, but it would be good to be more clear and specific about sizes.

*This is now clarified in this section as well as in the introduction.*

12. Line 355 mentions two "major autosomal translocations", but no details or references are given.

*These results are reported in the Results section Translocations to the W-SDR and palindromic repeats (lines 163-174 of original submission). We have added “(from Chr17 and Chr19)” to the specified line in the Discussion to clarify this point.*

13. When mentioning Arabidopsis thaliana, it whould be given its full name the first time it appears.

*Done.*

**Second round of review**

**Reviewer 2**

The revised version of this manuscript is still very badly written, with numerous things left vague, and it therefore still requires careful revision throughout. The new text, with some details that were not previously clear, now raises further puzzles, and reveals some errors in the biological concepts mentioned, concerning sex chromosomes and evolution more generally.

1.     The Abstract suggests that a question studied is "how coding sequence conservation is maintained in the absence of homologous recombination". However, it is well documented that purifying selection can maintain sequences with important functions, even in non-recombining regions, so the meaning is completely unclear here. A similar suggestion of a mystery where there is none appears in line 22.

2.     Statements about evolutionary times and distances are vague.

a.     Line 18 mentions closely related species without any quantification of how close they are. Synonymous site divergence estimates should be provided, with details about the number of sequences from which the estimates were derived, and their sizes. Line 35 mentions 'ancient' ones without being clear what the difference is.
b.     Line 175 mentions dS values (without explaining what they are). They seem to be synonymous site divergence estimates between 151 pairs of Salix "Z-degenerate" genes (an unclear category, as explained below), for comparison between estimates between species. It is not explained why PAML was used to estimate these simple pairwise divergence values.
c.     Line 255 mentions 'millions of years' without any explanation of where this comes from. If the synonymous site divergence estimates are being used, with a molecular clock, these need to be specified, with references for the clock data. The estimates between species are included in order to be able to infer a recent origin of the sex-linked region, even in Salix, where it includes around half of the chromosome.
d.     The statement that (in my revised version) fully sex-linked "W and Z alleles … have dS values similar to those for orthologous autosomal genes from S. purpurea and S. suchowensis" is unclear. I think the authors mean that the inter-species divergence is similar for autosomal and sex-linked genes, but I cannot be sure.

3.     Line 29: it is not correct that sex chromosomes "contain [meaning carry] the genes that confer sex-specific traits". Sex differences are controlled by genes on many chromosomes, and the sex chromosomes may carry (but not in species with sex chromosome-autosome balance systems) the master sex-determining locus, with downstream effects on other genes that may be involved in such differences. It is also not true that they are "unique", as other polymorphisms involving non-recombining regions exist (including fungal mating-type loci, some plant self-incompatibility loci, and some bird mating plumage types, among others). These share the two features mentioned, different 'versions' of the region in question occurring in different phenotypes, and absence of recombination, leading to similar consequences for evolution of these genome regions.
4.     Line 37: 'animal sex chromosomes' are mentioned without being clear how many animals have been examined, or what kind. I believe that this statement might be based on 2 mammals. Are the same features seen in insect sex chromosomes? If not, one surely cannot say 'animals' in this way.
5.     The terminology 'male-specific' is used to mean Y-linked. Of course, Y linkage can be detected through detection of male-specific sequences/variants, but the terms are not interchangeable, and the text ought to use the appropriate one. 'Y-linked' is also preferable to 'on the Y chromosome' when writing about species that don't have a well-defined Y chromosome, meaning at least a multi-gene Y-linked region, or a heteromorphic chromosome or chromosome region.
6.     The concept that XY and ZW systems might have the same underlying sex determination mechanism appears several times (e.g. line 56, 66 and others), but it is never clearly explained how this might work. It seems implausible, but if the authors want to suggest this, they need to explain it, and how it could work with the different dominance requirements of the two systems.
7.     The study provides inferred Z and W haplotypes of the chromosome that carries the sex-determining locus of the study species. This is a valuable achievement, and it appears to confirm that the species has a multi-gene W-linked region, despite the lack of W/Z heteromorphism. However, the results are presented in a manner that makes them difficult to follow.  I think that the main conclusion is that the fully W-linked region is estimated to be 6.4 Mb of chromosome 15, versus 4.1 Mb for the homologous fully Z-linked region; the rest of the chromosome consists of partially sex-linked region, or pseudo-autosomal regions (PARs), whose sizes are estimated to be 6.5 and 2.7 Mb. A diagram would be helpful, rather than the rambling text on page 7, which specifies the total size of chromosome 15 — this is confusing, because the W and Z versions have different sizes. With a diagram, the text on p. 7 can be made much shorter, and would also be clearer. In addition, the bottom part of the page, which describes problems encountered with the ws8 contig in detail, could be moved to a Supplementary text, to avoid interrupting the main points being outlined here. Figure 1 does show a diagram, but only of the W, and the size estimates of the PARs are not clear.

8.     The terminology used is confusing. The term W-SDR is introduced and defined in line 127, but not consistently used. At times, it seems as if terms like 'Chr15W' sometimes means the entire chromosome, and sometimes mean just the fully sex-linked, W-SDR part. The latter would seem to be the correct meaning. Page 9 also uses 'Chr15W SDR' and the terms W- and Z-SDR, which would be unnecessary if one used Chr15W and Chr15Z. The terminology should be explained clearly and then used consistently, to avoid difficulties for readers.

With a shortened version of this text, it would then be easy to explain that the larger size of the fully W-linked region that the corresponding Z region might be accounted for by its greater repeat content, motivating the study of transposable elements (rather than mentioning these after the gene density information, which makes it sound as if there is something different about the genes, when in fact the difference is simply in the non-genic content). The top of p. 8 mentions gene densities, and Table 1 shows these, plus densities of two types of transposable elements (the remark that "all 18 chromosomes are included" is unclear — should it say "all 18 autosomes …"?). It is unclear what "SDR" refers to in this table. It should be the estimate for the fully W-linked region, and this should be explicitly stated. Also, the word "frequency" is not correct, if the numbers shown are actually numbers of items per 100 kb.

9.     A strange finding mentioned in lien 148 is that the W-linked region (W-SDR) included many more genes than the corresponding Z-linked region. This is the opposite of what one would expect if there is a multi-gene W-linked region without recombination that would be expected to undergo genetic degeneration. Lines 163 onwards explain the statement in line 155 that the analysis to compare the two was restricted to the female (presumably meaning the genome sequences obtained from the female individual). It is not clear to me why this simplifies the analysis, as one would expect the Z sequence from the homozygous male to be most reliable.

I suspect that the authors are trying to explain that the expectation of lower numbers of genes in the W- than the Z-linked region suggested to them that some W-linked genes might have become amplified (as has been observed in other species). It would be helpful to mention briefly in the Introduction what is currently known the mammalian ampliconic genes, and whether these are "in situ" duplications of genes ancestrally present on the chromosome that evolved into the XY pair, or genes not ancestrally present that moved to the X or Y during sex chromosome evolution, or some of each kind. Many readers will not know these facts/inferences. Page 16 does state that "ampliconic sequences on the human Y chromosome were acquired through transpositions from diverse sources, and then amplified", but still does not make clear whether the meaning is "all ampliconic sequences on the human Y…". This is related to the paragraph on p. 18 discussing the small number of palindromic regions inferred in the Salix W-linked region, and the material could be integrated in one place.

However, the text really does not make this clear, and it requires revision so that readers can understand the points. The text continues, going on to discuss degeneration (and returns to the added genes only on p. 11), and uses the term "Z-degenerate genes" confusingly (to this reader). The text says that these refer to "single copy mutual best hits between the W-SDR and Z-SDR", which sounds like non-degenerated genes (termed "ancestral" in analyses of mammal XY chromosomes, for exampleby Sayres, M., & Makova, K. (2013. Gene survival and death on the human Y chromosome. Molecular Biology and Evolution, 30(4), 781-787. doi:10.1093/molbev/mss267). The term "X-degenerate" is used in the mammalian XY system to mean genes present on the X, but not found on the Y.

The analysis of gene contents, with inferences about gene movements, is not clearly enough described to tell whether it is convincing, and the conclusion about degeneration is left unclear, even though earlier analyses used depth of coverage, which presumably suggests degeneration of the W-linked region. I was left confused. The analysis surely needs to be directed towards assessing which genes are what are termed "ancestral", as defined above, and which are added genes. Genes in the first category are of interest with respect to degeneration, which cannot be determined without separating them from other categories of sequences.

10.     Further confusion arises when the 'intercross map' is mentioned in line 99, as it is not until much later (line 499) that it is explained that this is described in a different paper.

11.     The expectation in line 276 is not explained.

12.     The Discussion can be shortened by omitting unnecessary repeats and speculations (several are outlined below). It is unclear to me whether there is actual evidence for gene conversion, or merely evidence that the palindrome regions are more similar than expected. The analysis to show that they are more similar than expected is not clearly enough explained to be convincing that gene conversion is occurring. Line 256 simply mentions that conversion is "likely". Have selective constraints in essential sequences been excluded?

The new material starts in line 305, though this paragraph is confusing, and doesn't convey a clear meaning. I explained above why it seems unlikely that ZW and XY systems could have the same sex-determining system. Perhaps the authors are trying to suggest that Salix and Populus have the same sex-determining genes, despite the different dominance relationships. This is certainly not clearly conveyed, and it remains unclear what really solid evidence exists for a genome region on the sex chromosomes of species in both genera. The Salix sex-linked region is extensive, containing many sequences, and the finding that some of them are also present in the Populus region is not evaluated adequately in relation to the sizes of the two regions, and presence of specific single-copy genes.

Lines 334 onwards are another unnecessary repeat, and so are lines 393 onwards. Reasons to suspect "that the multi-copy genes in the S. purpurea palindrome might play important roles in sex determination and/or sex dimorphism" are mentioned, but not actually explained.

The conclusion that no strata could be found in Salix could be shortened, just mentioning the previous same conclusion and the alignment difficulties. The next paragraph can also be shortened by omitting unnecessary repetition. The passage about X chromosome palindromes might be better in the Introduction, to make clear that recombining regions can also have them.

13.     The next paragraph is also unclear. 'Autosomal translocations' may mean 'sex-chromosome-autosome translocations, and the authors are presumably trying to argue the case that the Salix and Populus genomes share chunks on their respective sex chromosomes. Later, in line 375, the phrase seems to mean transpositions into the W-linked region. The wording should be checked and used consistently and correctly.

14.     A paragraph on p. 18 discussed gene movements, suggesting that transposable elements led to transpositions of genes into the fully W-linked region. As there is no evidence for this in Salix, this speculation should be shortened and mentioned briefly along with the other discussion of the palindromes.

15.     The conclusions section is unnecessary, and merely repeats what has already been said. It should be removed, and space given to explain important things clearly.

Minor points
1.     In line 7, the word 'facilitate' is used when the authors merely mean that 'leads to'. The suggestion of adaptation should be removed.
2.     Line 14 should read "span" (not "over")
3.     Line 199: I think the authors mean 'identity", not 'conservation'. The same information is repeated in line 209, I think.
4.     Lines 259 and 260 use 'polymorphisms' incorrectly when substitutions between species are meant.

**Authors Response**

**Point-by-point responses to the reviewers’ comments:**

The revised version of this manuscript is still very badly written, with numerous things left vague, and it therefore still requires careful revision throughout. The new text, with some details that were not previously clear, now raises further puzzles, and reveals some errors in the biological concepts mentioned, concerning sex chromosomes and evolution more generally.

1. The Abstract suggests that a question studied is "how coding sequence conservation is maintained in the absence of homologous recombination". However, it is well documented that purifying selection can maintain sequences with important functions, even in non-recombining regions, so the meaning is completely unclear here. A similar suggestion of a mystery where there is none appears in line 22.

*We agree that purifying selection can still function in regions with suppressed recombination, albeit with reduced efficiency due to Hill-Robertson effects. We have revised the sentence as follows to make it more accurate: " Plant sex chromosomes are less well understood, and in particular it is not clear if palindromic repeats play a role in maintaining sequence conservation in the absence of homologous recombination."*

2. Statements about evolutionary times and distances are vague.

a. Line 18 mentions closely related species without any quantification of how close they are. Synonymous site divergence estimates should be provided, with details about the number of sequences from which the estimates were derived, and their sizes. Line 35 mentions 'ancient' ones without being clear what the difference is.

*As reported on line 175 and depicted in Figure 3, "the dS between syntenic genes on Chr01 for S. purpurea and S. suchowensis was 0.045±0.085". This is the basis for calling the species "closely-related.". We have decided not to specify this on line 18 since it is awkward to cite such details in the abstract. The first place where S. suchowensis is mentioned in the body of the manuscript is the line cited above, thereby objectively quantifying the degree of relatedness at the outset, as requested by the reviewer.*

*We agree that the text on line 35 was too vague and we have revised for accuracy by dropping the term "ancient" since the observation really applies to all sex chromosomes with large amounts of heterochromatic and repetitive sequence.*

b. Line 175 mentions dS values (without explaining what they are). They seem to be synonymous site divergence estimates between 151 pairs of Salix "Z-degenerate" genes (an unclear category, as explained below), for comparison between estimates between species. It is not explained why PAML was used to estimate these simple pairwise divergence values.

*The dS estimates are explained in the Methods section (line 610). We agree that it is awkward to have the Methods section at the end of the manuscript, and we recognize that many readers will not skip to that section for clarification of details when reading the Results section. We have therefore followed the reviewer's recommendation and added some clarifying text throughout the Results section. In this case, we specified that dS refers to divergence of syntenic gene pairs on the Z and W chromosomes, which is a standard approach for detecting evolutionary strata.*

*To improve clarity, we have also adopted the subscript notation used in the original publication describing these estimates [1].*

*We used PAML for these estimates in response to a request in the original review of this manuscript to provide confidence intervals for these estimates. PAML is the most commonly-used software for these calculations, and it provides the needed confidence intervals. This also prompted our switch from KS to dS, since the confidence intervals for the former estimates are not provided by PAML.*

c. Line 255 mentions 'millions of years' without any explanation of where this comes from. If the synonymous site divergence estimates are being used, with a molecular clock, these need to be specified, with references for the clock data. The estimates between species are included in order to be able to infer a recent origin of the sex-linked region, even in Salix, where it includes around half of the chromosome.

*The full explanation for the origin of these estimates, including a citation for the molecular clock, occurs in the previous paragraph in the manuscript (lines 241-245 of the original submission). We have clarified this by adding "based on the retrotransposon analysis."*

d. The statement that (in my revised version) fully sex-linked "W and Z alleles … have dS values similar to those for orthologous autosomal genes from S. purpurea and S. suchowensis" is unclear. I think the authors mean that the inter-species divergence is similar for autosomal and sex-linked genes, but I cannot be sure.

*We agree that this text was potentially confusing, so we have simplified the comparison. We are now simply pointing out that the S. purpurea SDR is relatively young, and that it was likely established before divergence of S. purpurea and S. suchowensis, but well after the divergence of Salix from Populus: "The SDR of S. purpurea also appears to be established relatively recently, based on the low level of divergence between homologs on Chr15W and Chr15Z in the nonrecombining SDR. Furthermore, given that the SDR is located in approximately the same portion of Chr15 in both S. purpurea and S. suchowensis, and both have ZW systems [21,23], it is reasonable to assume that the SDR became established in this lineage prior to divergence of these two species, but well after divergence from Populus, which has the SDR on Chr19."*

3. Line 29: it is not correct that sex chromosomes "contain [meaning carry] the genes that confer sex-specific traits". Sex differences are controlled by genes on many chromosomes, and the sex chromosomes may carry (but not in species with sex chromosome-autosome balance systems) the master sex-determining locus, with downstream effects on other genes that may be involved in such differences. It is also not true that they are "unique", as other polymorphisms involving non-recombining regions exist (including fungal mating-type loci, some plant selfincompatibility loci, and some bird mating plumage types, among others). These share the two features mentioned, different 'versions' of the region in question occurring in different phenotypes, and absence of recombination, leading to similar consequences for evolution of these genome regions.

*We have revised this sentence to make it more accurate: "Sex chromosomes are fascinating because they carry genes that confer or control sex-specific traits".*

4. Line 37: 'animal sex chromosomes' are mentioned without being clear how many animals have been examined, or what kind. I believe that this statement might be based on 2 mammals. Are the same features seen in insect sex chromosomes? If not, one surely cannot say 'animals' in this way.

*Excellent point. We have changed "animal" to "mammalian" in this sentence. Palindromes have been observed in many mammalian sex chromosomes (human, gorilla, ape, chimpanzee, rhesus macaque, mouse, and pig), so we think this statement is justified.*

5. The terminology 'male-specific' is used to mean Y-linked. Of course, Y linkage can be detected through detection of male-specific sequences/variants, but the terms are not interchangeable, and the text ought to use the appropriate one. 'Y-linked' is also preferable to 'on the Y chromosome' when writing about species that don't have a well-defined Y chromosome, meaning at least a multi-gene Y-linked region, or a heteromorphic chromosome or chromosome region.

*We agree that our use of the terms 'male-specific' and 'female-specific' was imprecise. We have removed all uses of these terms throughout the manuscript and replaced them with terms that made it clear when we were referring to the W haplotype or the Y haplotype, or to W-linked or Y-linked markers. We have reserved the term femalespecific to refer to stretches of sequence that only occur in the W haplotype.*

6. The concept that XY and ZW systems might have the same underlying sex determination mechanism appears several times (e.g. line 56, 66 and others), but it is never clearly explained how this might work. It seems implausible, but if the authors want to suggest this, they need to explain it, and how it could work with the different dominance requirements of the two systems.

*We agree that it is unlikely that the exact mechanisms of sex determination would be the same in XY and ZW systems. However, it is not unreasonable to assume that the two systems would contain some shared underlying features, since the developmental pathways of closely-related XY and ZW species are sometimes quite similar (e.g., in Populus and Salix). In fact, a prominent model of the transitions between XY and ZW systems includes transposition of the sex determining locus from a sex chromosome to an autosome, and subsequent conversion of the autosome to a new sex chromosome [2]. However, we agree with the reviewer that it is unlikely that a sex-determining factor in an XY chromosome could play an identical role in a ZW system. We have therefore revised the text to reflect this uncertainty: " More recently it has been shown that some species of Silene have ZW sex determination systems, though it remains unclear if there are commonalities in the underlying mechanisms of sex determination in XY and ZW species [20]." and "We also demonstrate that homologous genes occur in the Salix and Populus SDRs, suggesting that there may be some overlap in the underlying mechanisms of sex determination in this family."*

7. The study provides inferred Z and W haplotypes of the chromosome that carries the sexdetermining locus of the study species. This is a valuable achievement, and it appears to confirm that the species has a multi-gene W-linked region, despite the lack of W/Z heteromorphism. However, the results are presented in a manner that makes them difficult to follow. I think that the main conclusion is that the fully W-linked region is estimated to be 6.4 Mb of chromosome 15, versus 4.1 Mb for the homologous fully Z-linked region; the rest of the chromosome consists of partially sex-linked region, or pseudo-autosomal regions (PARs), whose sizes are estimated to be 6.5 and 2.7 Mb. A diagram would be helpful, rather than the rambling text on page 7, which specifies the total size of chromosome 15 — this is confusing, because the W and Z versions have different sizes. With a diagram, the text on p. 7 can be made much shorter, and would also be clearer.

*We have revised this section of the text to make the results simpler and clearer. We also added a diagram (Fig. S3) that shows the relative sizes of the components of Chr15W and Chr15Z. Also, Figure 1 is drawn to scale and contains the coordinates of the PARs and the SDR of the W chromosome.*

In addition, the bottom part of the page, which describes problems encountered with the ws8 contig in detail, could be moved to a Supplementary text, to avoid interrupting the main points being outlined here. Figure 1 does show a diagram, but only of the W, and the size estimates of the PARs are not clear.

*We have removed the text about the apparent chimeric nature of ws9. We agree that it detracted from the focus of this section, and did not contribute important information. We have also added a supplementary figure to make the relative sizes of the Z and W chromosome components more clear (Fig. S3).*

8. The terminology used is confusing. The term W-SDR is introduced and defined in line 127, but not consistently used. At times, it seems as if terms like 'Chr15W' sometimes means the entire chromosome, and sometimes mean just the fully sex-linked, W-SDR part. The latter would seem to be the correct meaning. Page 9 also uses 'Chr15W SDR' and the terms W- and Z-SDR, which would be unnecessary if one used Chr15W and Chr15Z. The terminology should be explained clearly and then used consistently, to avoid difficulties for readers.

*We agree that our rather loose use of this terminology could cause confusion. We have edited the manuscript so that the W haplotype is always referred to as the W-SDR and the corresponding region on the Z chromosome is always referred to as the Z-SDR. The PAR portions of the chromosomes are unphased and identical in sequence.*

With a shortened version of this text, it would then be easy to explain that the larger size of the fully W-linked region that the corresponding Z region might be accounted for by its greater repeat content, motivating the study of transposable elements (rather than mentioning these after the gene density information, which makes it sound as if there is something different about the genes, when in fact the difference is simply in the non-genic content). The top of p. 8 mentions gene densities, and Table 1 shows these, plus densities of two types of transposable elements (the remark that "all 18 chromosomes are included" is unclear — should it say "all 18 autosomes …"?). It is unclear what "SDR" refers to in this table. It should be the estimate for the fully Wlinked region, and this should be explicitly stated. Also, the word "frequency" is not correct, if the numbers shown are actually numbers of items per 100 kb.

*This is an excellent point. We have revised Table 1 so that it is clear that we are comparing the W-SDR, the Z-SDR, the PARs, and the autosomes. We are now reporting the total extent of genes, all repeats, and Gypsy and Copia LTR retrotransposons in each of these regions, as well as the percent of the regions covered by these categories. This is a much better way to highlight the differences among these portions of the genome, and we thank the reviewer for this suggestion.*

*We have chosen not to reorganize as suggested because the purpose of the LTR analysis was to determine the relative timing of the transpositions that led to the formation of the palindromic repeats. This is most logically covered after the description of the palindromes.*

9. A strange finding mentioned in lien 148 is that the W-linked region (W-SDR) included many more genes than the corresponding Z-linked region. This is the opposite of what one would expect if there is a multi-gene W-linked region without recombination that would be expected to undergo genetic degeneration.

*We agree that this is the generally accepted model for sex chromosome evolution. In fact, we would like to suggest that our manuscript provides a valuable data point since it is quite clear that the W-SDR has accumulated more genes than the Z-SDR following cessation of recombination, in part due to transpositions from the autosomes. This is one of the most important findings of our paper, and we have further highlighted it in the revised abstract, discussion, and conclusions.*

Lines 163 onwards explain the statement in line 155 that the analysis to compare the two was restricted to the female (presumably meaning the genome sequences obtained from the female individual). It is not clear to me why this simplifies the analysis, as one would expect the Z sequence from the homozygous male to be most reliable.

*The Z sequence from the homozygous male could not be readily phased to a single haplotype due to a general lack of sequence divergence between the Z haplotypes. We therefore focused our efforts on phasing the W and Z haplotypes of the female. The male reference genome assembly is therefore an amalgamation of two Z haplotypes (as are most reference genomes), and does not represent an actual haplotype that occurs in nature. In contrast, because the female W-SDR and Z-SDR are largely phased, these can be directly compared without the potentially confounding effects of artifacts from the assembly process. We validated this approach by comparing the size and gene content of the Z-SDR regions of the male and female, and they were largely concordant, as stated in the manuscript.*

I suspect that the authors are trying to explain that the expectation of lower numbers of genes in the W- than the Z-linked region suggested to them that some W-linked genes might have become amplified (as has been observed in other species). It would be helpful to mention briefly in the Introduction what is currently known the mammalian ampliconic genes, and whether these are "in situ" duplications of genes ancestrally present on the chromosome that evolved into the XY pair, or genes not ancestrally present that moved to the X or Y during sex chromosome evolution, or some of each kind. Many readers will not know these facts/inferences. Page 16 does state that "ampliconic sequences on the human Y chromosome were acquired through transpositions from diverse sources, and then amplified", but still does not make clear whether the meaning is "all ampliconic sequences on the human Y…". This is related to the paragraph on p. 18 discussing the small number of palindromic regions inferred in the Salix W-linked region, and the material could be integrated in one place. However, the text really does not make this clear, and it requires revision so that readers can understand the points.

*We have added text to the Introduction to describe the origin and evolution of ampliconic genes on human Y chromosomes.*

The text continues, going on to discuss degeneration (and returns to the added genes only on p. 11), and uses the term "Z-degenerate genes" confusingly (to this reader). The text says that these refer to "single copy mutual best hits between the W-SDR and Z-SDR", which sounds like nondegenerated genes (termed "ancestral" in analyses of mammal XY chromosomes, for exampleby Sayres, M., & Makova, K. (2013. Gene survival and death on the human Y chromosome. Molecular Biology and Evolution, 30(4), 781-787. doi:10.1093/molbev/mss267). The term "Xdegenerate" is used in the mammalian XY system to mean genes present on the X, but not found on the Y.

*We certainly agree with the reviewer that the term "Z-degenerate genes" is confusing. We have decided to use "Z-W homologs" instead. Unfortunately, the term X-degenerate was coined in the original publication describing Human Y chromosome gene content to refer to "single copy gene or pseudogene homologues of x-linked genes" (Figure 4 in Skaletsky et al. 2003). This term has been widely applied in the animal sex chromosome literature using this same definition. We have therefore pointed out that "Z-W homologs" is analogous to "X-degenerate genes".*

The analysis of gene contents, with inferences about gene movements, is not clearly enough described to tell whether it is convincing, and the conclusion about degeneration is left unclear, even though earlier analyses used depth of coverage, which presumably suggests degeneration of the W-linked region. I was left confused. The analysis surely needs to be directed towards assessing which genes are what are termed "ancestral", as defined above, and which are added genes. Genes in the first category are of interest with respect to degeneration, which cannot be determined without separating them from other categories of sequences.

*This is an excellent suggestion. Our previous analysis assumed that genes that were present on the W but absent on the Z had been transposed from autosomes IF those genes had a very close paralog on the autosome in S. purpurea. To improve this analysis, we checked Chr15 of P. trichocarpa (as an outgroup) to identify genes that may have been lost from the Z chromosome but retained on the W. We also repeated this analysis for all Z-linked genes that were absent from the W. We refer to these as "ancestral genes" and we have incorporated these results into Fig. 2. We discovered a total of 18 of these genes on the W chromosome and 20 on the Z chromosome. There are still 40 genes that originated from autosomal transpositions on the W, compared to only 11 on the Z, so it is still apparent that autosomal transpositions are an important contributor to sex chromosome evolution in this species. Intrachromosomal duplication of W-linked genes accounts for much of the rest of the difference in gene content (44 on the W compared to 7 on the Z). We have clarified all of this in the Results and Methods sections.*

10. Further confusion arises when the 'intercross map' is mentioned in line 99, as it is not until much later (line 499) that it is explained that this is described in a different paper.

*We have clarified that this refers to the map that was used in the original genome assembly, and we have cited the published paper that more fully describes that work.*

11. The expectation in line 276 is not explained.

*Those genes only occur on the W chromosome, so we did not expect to see expression in males, which lack the W. However, we agree that this was unclear, so we deleted "As expected." It was not a crucial point, and it detracted from clarity.*

12. The Discussion can be shortened by omitting unnecessary repeats and speculations (several are outlined below). It is unclear to me whether there is actual evidence for gene conversion, or merely evidence that the palindrome regions are more similar than expected. The analysis to show that they are more similar than expected is not clearly enough explained to be convincing that gene conversion is occurring. Line 256 simply mentions that conversion is "likely". Have selective constraints in essential sequences been excluded?

*We followed a standard analysis to present evidence of gene conversion. We showed that in the case of the response regulator gene family, all four copies in the palindrome are completely identical in sequence, yet these genes are divergent from their multi-copy orthologs in Salix suchowensis. This is exactly how gene conversion was demonstrated on mammalian Y chromosomes, for example [4]. This is one of the most important findings of the manuscript, and we have revised the text to more clearly explain the approach, and to emphasize its importance.*

The new material starts in line 305, though this paragraph is confusing, and doesn't convey a clear meaning. I explained above why it seems unlikely that ZW and XY systems could have the same sex-determining system. Perhaps the authors are trying to suggest that Salix and Populus have the same sex-determining genes, despite the different dominance relationships. This is certainly not clearly conveyed, and it remains unclear what really solid evidence exists for a genome region on the sex chromosomes of species in both genera. The Salix sex-linked region is extensive, containing many sequences, and the finding that some of them are also present in the Populus region is not evaluated adequately in relation to the sizes of the two regions, and presence of specific single-copy genes.

*As explained in the opening sentence of the cited paragraph, "Our W chromosome assembly has helped explain why sex association analyses sometimes detect multiple unlinked loci on different chromosomes [5–7]." The rest of the paragraph explains that these multiple peaks are caused by incorrect sequence read alignments to the original sequence locations in cases where the transposed sequences are not included in the reference genome. We included this paragraph because the cited papers reported the multiple peaks of sex association without properly explaining their cause. Nevertheless, we agree that this point is marginally important, so we have removed this paragraph from the discussion, since it distracts from our main findings.*

Lines 334 onwards are another unnecessary repeat, and so are lines 393 onwards. Reasons to suspect "that the multi-copy genes in the S. purpurea palindrome might play important roles in sex determination and/or sex dimorphism" are mentioned, but not actually explained.

The conclusion that no strata could be found in Salix could be shortened, just mentioning the previous same conclusion and the alignment difficulties. The next paragraph can also be shortened by omitting unnecessary repetition. The passage about X chromosome palindromes might be better in the Introduction, to make clear that recombining regions can also have them.

*We very much appreciate these suggestions for reorganization of the Discussion, and we have now reorganized it to have a more logical flow of ideas.*

13. The next paragraph is also unclear. 'Autosomal translocations' may mean 'sexchromosome-autosome translocations, and the authors are presumably trying to argue the case that the Salix and Populus genomes share chunks on their respective sex chromosomes. Later, in line 375, the phrase seems to mean transpositions into the W-linked region. The wording should be checked and used consistently and correctly.

*We have changed the terminology to use the more accurate term "transposition" rather than translocation to describe the movement of genes from the autosomes to the WSDRs. This is especially appropriate since we have some evidence that this transposition is in fact mediated by transposable elements. We have also tried to clarify the point that the shared genes between the Salix W-SDR and the Populus SDR suggest some commonalities in the underlying mechanisms of sex determination, since an orthologous gene (based on mutual best blast hits) is associated with sex in both species. That gene (RR9) shows evidence of gene conversion in S. purpurea, and it is the only gene in the P. balsamifera genome that shows sex-biased methylation [8]. We have tried to build this case more clearly in the revised Discussion.*

14. A paragraph on p. 18 discussed gene movements, suggesting that transposable elements led to transpositions of genes into the fully W-linked region. As there is no evidence for this in Salix, this speculation should be shortened and mentioned briefly along with the other discussion of the palindromes.

*The presence of unique target site duplications and LTRs flanking some of the palindrome arms would generally be considered good evidence of prior transposition. We have clarified this in the Results and Discussion.*

15. The conclusions section is unnecessary, and merely repeats what has already been said. It should be removed, and space given to explain important things clearly.

*This section is required by Genome Biology, and a quick survey of recently-published papers did not turn up any that excluded it. We have therefore decided to keep it, but have revised it to minimize unnecessary repetition and highlight our main findings and their implications.*

Minor points

1. In line 7, the word 'facilitate' is used when the authors merely mean that 'leads to'. The suggestion of adaptation should be removed.

*Edited as suggested.*

2. Line 14 should read "span" (not "over")

*Edited as suggested.*

3. Line 199: I think the authors mean 'identity", not 'conservation'. The same information is repeated *in line 209, I think.*

*Edited as suggested. The text on line 209 is not repetitive. It refers to gene sequences in all of the palindrome arms, which the text in the prior section refers to the entire stretch (coding and noncoding) of one of the palindromes.*

4. Lines 259 and 260 use 'polymorphisms' incorrectly when substitutions between species are meant.

*Edited as suggested.*

1. Yang Z, Nielsen R. Estimating Synonymous and Nonsynonymous Substitution Rates Under Realistic Evolutionary Models. Mol. Biol. Evol. 2000;17:32–43.

2. van Doorn GS, Kirkpatrick M. Turnover of sex chromosomes induced by sexual conflict. Nature. 2007;449:909–12.

3. Skaletsky H, Kuroda-Kawaguchi T, Minx PJ, Cordum HS, Hillier L, Brown LG, et al. The male-specific region of the human Y chromosome is a mosic of discrete sequence classes. Nature. 2003;423:825–37.

4. Rozen S, Skaletsky H, Marszalek JD, Minx PJ, Cordum HS, Waterston RH, et al. Abundant gene conversion between arms of palindromes in human and ape Y chromosomes. Nature. 2003;423:873–6.

5. Zhou R, Macaya-Sanz D, Rodgers-Melnick E, Carlson CH, Gouker FE, Evans LM, et al. Characterization of a large sex determination region in Salix purpurea L. (Salicaceae). Mol. Genet. Genomics. 2018;293:1437–52.

6. Geraldes A, Hefer CA, Capron A, Kolosova N, Martinez-Nuñez F, Soolanayakanahally RY, et al. Recent Y chromosome divergence despite ancient origin of dioecy in poplars (Populus). Mol. Ecol. 2015;

7. Pucholt P, Hallingbäck HR, Berlin S. Allelic incompatibility can explain female biased sex ratios in dioecious plants. BMC Genomics. 2017;18:251.

8. Bräutigam K, Soolanayakanahally R, Champigny M, Mansfield S, Douglas C, Campbell MM, et al. Sexual epigenetics: gender-specific methylation of a gene in the sex determining region of Populus balsamifera. Sci. Rep. 2017;7:45388.

**Third round of review**

**Reviewer 2**

The revised version of this manuscript is improved again, but still poorly written and hard to understand in places. It is up to the editors to evaluate my major comments below, and decide whether the writing is unacceptably vague, or whether the vague ideas are permissible in a scientific paper.

The evolutionary time estimates are still unclear. The revision states in response to comment c: "We have shown that the palindromes are likely to be millions of years old based on the retrotransposon analysis", but I still could not find a clear account of how the estimates were obtained, and how that analysis relates to the dS values that are also mentioned.


I hate to nit-pick, but the revised Introduction now gives the impression that sex chromosome heteromorphism is absent in papaya (which isn't the case, though the X-Y differences are confined to a small bit of a chromosome, so are hard to detect by conventional cytogenetics), and fails to make clear the pronounced heteromorphism in some Silene species. feel that it is important to communicate accurately the science, as currently understood, and I don't really think that the revised text achieves this.

I also I don't really think that the revised sentence "Sex chromosomes are
fascinating because they carry genes that confer or control sex-specific traits" is accurate. I feel that readers will find the end of this sentence too vague to tell what the authors are trying to say (and, if it's not too vague, then it's not accurate, because sex-specific traits are controlled by the genetic sex interacting with control of autosomal genes). Moreover, some people may feel that sex chromosomes are interesting because (unlike most genome regions) they do not recombine, so the statement is not really accurate. Why not simply say that the evolution of large non-recombining regions is interesting, and the involvement of mutations that lead to sex-specific traits in the evolution of such fully sex-linked regions is currently not understood.

The ms and responses to point 6 still do not explain how XY and ZW systems might have the same underlying sex
determination mechanism, and the authors' thinking seems muddled. Of course, the downstream developmental
pathways leading to male and female parts of closely-related XY and ZW plants will be similar, but the actual control of whether individuals are male or female cannot be the same in those systems. At the very least, one must recognize that maleness is dominant in XY and recessive in ZW systems. If the X of XY systems carries a recessive male-sterility factor, it cannot be present in the Z of a ZW system, because males are ZZ. The authors agree with the reviewer that it is unlikely that a sex-determining factor in an XY chromosome could play an identical role in a ZW system, and it seems unhelpful to retreat into vague statements like the proposed "it remains unclear if there are commonalities in the underlying mechanisms of sex determination in XY and ZW species" and argue that the presence of " homologous genes .. in the Salix
and Populus SDRs, suggests that there may be some overlap in the underlying mechanisms of sex determination in this family." These statements are too vague to be evaluated. Speculation has its place, but at the very least, should offer plausible mechanisms, or ideas for empirical testing.

Can the authors explain the results on p. 5, where alternative haplotypes total 72.4 Mb in the female assembly, versus 149 Mb for the male one, yet the 19 chromosome assemblies are the same in the two sexes. Do these phased regions represent  only a part of the complete genome assembly, presumably disproportionately represent the large fully sex-linked region? This is not made clear.

Minor comments
The statement "Plant sex chromosomes are less well understood" is vague and sweeping. They are very well understood in several respects, in some species. The authors' point is simply that they have evidence for palindromic repeats in their Salix species, and it would be best simply to say that.

The ± signs for dS values should be explained (the responses suggest that they are 95% confidence intervals based on using PAML).

I think that the term "PAR" is used in line 102 but not defined until line 127. The sentence is confusiong and needs commas as follows
There are two pseudoautosomal regions (PARs), one at each end of Chr15W, with
indistinguishable sequences in the corresponding regions on Chr15Z.

Also GBS in line 117 is not defined.

I think it should be "absent from" (not absent on).

The text is still frequently less clear than it could be, For example
that the S. purpurea SDR is relatively
young, and that it was likely established before divergence of S. purpurea and S.
suchowensis, but well after the divergence of Salix from Populus: "The SDR of S.
purpurea also appears to be established relatively recently, based on the low level of
divergence between homologs on Chr15W and Chr15Z in the nonrecombining SDR.

Means simply: "The low divergence between homologs in the fully sex-linked region (i.e. between Chr15W and Chr15Z sequences) shows that the SDR of S. purpurea evolved recently, well after the divergence of Salix from Populus, although before divergence of S. purpurea and S. suchowensis, consistent with the ZW SDRs being located in approximately the same Chr15 region in both these species [21,23], whereas the "XY" SDR Populus is on Chr19".


The first time that "female-specific" is used, it would be good to define it. The revision has improved this aspect of the text, but it would be best to avoid any possible misunderstanding.

Vague terms like "main genome" should be clarified.

The text mentions that "The recently transposed genes are of particular interest because of the insights they can provide into the evolutionary history of the sex chromosome", but it is not explained what insights were actually obtained. The text around line 187 is not very clear, as "contain ws19" isn't explained — presumably it is from the Salix purpurea W-linked region, but the notation needs to be explained.

**Authors Response**

**Point-by-point responses to the reviewers’ comments:**

Reviewer #2

The revised version of this manuscript is improved again, but still poorly written and hard to understand in places. It is up to the editors to evaluate my major comments below, and decide whether the writing is unacceptably vague, or whether the vague ideas are permissible in a scientific paper.

The evolutionary time estimates are still unclear. The revision states in response to comment c: "We have shown that the palindromes are likely to be millions of years old based on the retrotransposon analysis", but I still could not find a clear account of how the estimates were obtained, and how that analysis relates to the dS values that are also mentioned.

*The methods for this analysis are described in the Methods section, (lines 692 to 699). In the previous version we did not explicitly say in that section that the time estimates were derived from the mutation rate reported for P. tremula. That information was provided in the Results section (line 279). We have included the same information in the Methods now to reduce confusion about the source of these estimates. This analysis is entirely independent from the d_S_ estimates, which are based on comparing homologs between the Z and W to estimate relative time since cessation or recombination, in comparison to dS between orthologous genes of S. suchowensis and P. trichocarpa. The principle of estimating time since transposition based on LTR substitutions is explained in reference [70], which we have added to the Methods section for clarity.*

I hate to nit-pick, but the revised Introduction now gives the impression that sex chromosome heteromorphism is absent in papaya (which isn't the case, though the X-Y differences are confined to a small bit of a chromosome, so are hard to detect by conventional cytogenetics), and fails to make clear the pronounced heteromorphism in some Silene species. feel that it is important to communicate accurately the science, as currently understood, and I don't really think that the revised text achieves this.

*We have revised this paragraph to remove the impression that Papaya is homomorphic, and to make it clear that Silene is heteromorphic.*

I also I don't really think that the revised sentence "Sex chromosomes are
fascinating because they carry genes that confer or control sex-specific traits" is accurate. I feel that readers will find the end of this sentence too vague to tell what the authors are trying to say (and, if it's not too vague, then it's not accurate, because sex-specific traits are controlled by the genetic sex interacting with control of autosomal genes). Moreover, some people may feel that sex chromosomes are interesting because (unlike most genome regions) they do not recombine, so the statement is not really accurate. Why not simply say that the evolution of large non-recombining regions is interesting, and the involvement of mutations that lead to sex-specific traits in the evolution of such fully sex-linked regions is currently not understood.

*We agree. We have revised the sentence so that it no longer makes the claim that sex chromosomes are fascinating, which is an unnecessarily subjective opinion.*

The ms and responses to point 6 still do not explain how XY and ZW systems might have the same underlying sex determination mechanism, and the authors' thinking seems muddled. Of course, the downstream developmental pathways leading to male and female parts of closely-related XY and ZW plants will be similar, but the actual control of whether individuals are male or female cannot be the same in those systems. At the very least, one must recognize that maleness is dominant in XY and recessive in ZW systems. If the X of XY systems carries a recessive male-sterility factor, it cannot be present in the Z of a ZW system, because males are ZZ. The authors agree with the reviewer that it is unlikely that a sex-determining factor in an XY chromosome could play an identical role in a ZW system, and it seems unhelpful to retreat into vague statements like the proposed "it remains unclear if there are commonalities in the underlying mechanisms of sex determination in XY and ZW species" and argue that the presence of " homologous genes .. in the Salix and Populus SDRs, suggests that there may be some overlap in the underlying mechanisms of sex determination in this family." These statements are too vague to be evaluated. Speculation has its place, but at the very least, should offer plausible mechanisms, or ideas for empirical testing.

*We respect the reviewer’s opinion, but we have chosen to retain this somewhat speculative argument in the manuscript. The fact that the same plausible sex determination gene occurs in the SDR of both P. trichocarpa and S. purpurea, despite the fact that the SDR occurs on different chromosomes in both species, is very strong circumstantial evidence that this gene is involved in sex determination in both species. In the Conclusions section of the manuscript, we acknowledge the conundrum raised by the reviewer and provide a possible explanation:*

*“it is currently unclear how the same gene is functioning in an XY system in Populus and a ZW system in Salix. It is possible that the W chromosome version acts as a dominant promoter of female function, while the Y version is a dominant suppressor of female function, based on the putative roles of cytokinin and the type A response regulators in female development in Arabidopsis.”*

*We believe this is a plausible and clear model for how ZW and XY systems could mediate sex determination through the same pathway, so we are quite confident that this is an appropriate line of speculation that is consistent with the data that we have presented in this manuscript.*

Can the authors explain the results on p. 5, where alternative haplotypes total 72.4 Mb in the female assembly, versus 149 Mb for the male one, yet the 19 chromosome assemblies are the same in the two sexes. Do these phased regions represent  only a part of the complete genome assembly, presumably disproportionately represent the large fully sex-linked region? This is not made clear.

*The alternative haplotypes are derived from regions of the genome in which the haplotypes are sufficiently diverged that they can be assembled as separate phased contigs. The large discrepancy is due to a preponderance of long contigs among the alternative haplotypes in the Fish Creek genome. There are 31 contigs that exceed 1 Mb in the Fish Creek alternative haplotypes, totaling 44.8 Mb in cumulative length. In contrast, there are only 4 haplotypes longer than 1 Mb in 94006, totaling only 5.8 Mb. The amount and quality of sequencing data used for these assemblies is quite comparable (see Additional File 1, Table , so the discrepancy is likely due to differences in the genomes of the sequenced individuals. There are several plausible explanations for why this might be the case. Most notably, clone 94006 was collected from a naturalized population of S. purpurea that likely has experienced substantial inbreeding. Indeed, we have observed long stretches of homozygosity in this genome. On the other hand Fish Creek is an offspring of clone 94006 and a male collected from a different population, so it is likely to be more outbred and therefore carry more divergent haplotypes that can be readily phased. However, determining exactly why the Fish Creek genome is somewhat more phased than 94006 would require another set of independent analyses, and it would detract from the core focus of this paper. We have therefore decided not to include this analysis for the current manuscript, in which we have restricted analysis of Fish Creek to corroboration of our Z chromosome assembly for 94006.*

Minor comments
The statement "Plant sex chromosomes are less well understood" is vague and sweeping. They are very well understood in several respects, in some species. The authors' point is simply that they have evidence for palindromic repeats in their Salix species, and it would be best simply to say that.

*We have removed that statement from the Abstract.*

The ± signs for dS values should be explained (the responses suggest that they are 95% confidence intervals based on using PAML).

*The ± refer to standard errors. We have included “SE” following the values to indicate this.*

I think that the term "PAR" is used in line 102 but not defined until line 127. The sentence is confusiong and needs commas as follows
There are two pseudoautosomal regions (PARs), one at each end of Chr15W, with
indistinguishable sequences in the corresponding regions on Chr15Z.

*Fixed.*

Also GBS in line 117 is not defined.

*Fixed.*

I think it should be "absent from" (not absent on).

*Fixed.*

The text is still frequently less clear than it could be, For example
that the S. purpurea SDR is relatively
young, and that it was likely established before divergence of S. purpurea and S.
suchowensis, but well after the divergence of Salix from Populus: "The SDR of S.
purpurea also appears to be established relatively recently, based on the low level of
divergence between homologs on Chr15W and Chr15Z in the nonrecombining SDR.

Means simply: "The low divergence between homologs in the fully sex-linked region (i.e. between Chr15W and Chr15Z sequences) shows that the SDR of S. purpurea evolved recently, well after the divergence of Salix from Populus, although before divergence of S. purpurea and S. suchowensis, consistent with the ZW SDRs being located in approximately the same Chr15 region in both these species [21,23], whereas the "XY" SDR Populus is on Chr19".

*We have reworded those sentences to improve clarity.*

The first time that "female-specific" is used, it would be good to define it. The revision has improved this aspect of the text, but it would be best to avoid any possible misunderstanding.

*Done*

Vague terms like "main genome" should be clarified.

*We added a definition in the first paragraph of the Results section.*

The text mentions that "The recently transposed genes are of particular interest because of the insights they can provide into the evolutionary history of the sex chromosome", but it is not explained what insights were actually obtained. The text around line 187 is not very clear, as "contain ws19" isn't explained — presumably it is from the Salix purpurea W-linked region, but the notation needs to be explained.

*We have reworded the introductory sentence to that paragraph to enhance clarity about what we expected to learn from the recently transposed genes. The phrase “contain ws19” doesn’t occur in the manuscript. Ws19 is simply a contig number, so we refer to it as “contig ws19” which should be unambiguous. The contig numbers appear in Fig. 1, which is cited immediately after that sentence, so this should be clear to the reader.*
